# Warming‐Induced Effects on Microbial Communities and Nitrogen Cycling Capacity in Tundra Litter Are Modulated by Herb Abundance and Litter Quality

**DOI:** 10.1111/gcb.70582

**Published:** 2025-11-12

**Authors:** Mathilde Jeanbille, Karina E. Clemmensen, Jaanis Juhanson, Anders Michelsen, Juha Alatalo, Elisabeth J. Cooper, Greg H. R. Henry, Annika Hofgaard, Robert D. Hollister, Ingibjörg S. Jónsdóttir, Kari Klanderud, Anne Tolvanen, Sara Hallin

**Affiliations:** ^1^ Department of Forest Mycology and Plant Pathology Swedish University of Agricultural Sciences Uppsala Sweden; ^2^ Department of Biology, Terrestrial Ecology Section University of Copenhagen Copenhagen Denmark; ^3^ Environmental Science Center Qatar University Doha Qatar; ^4^ Faculty of Biosciences, Fisheries and Economics, Department of Arctic and Marine Biology UiT ‐ The Arctic University of Norway Tromsø Norway; ^5^ Department of Geography The University of British Columbia Vancouver Canada; ^6^ Norwegian Institute for Nature Research Trondheim Norway; ^7^ Grand Valley State University Allendale Michigan USA; ^8^ Life and Environmental Sciences University of Iceland Reykjavik Iceland; ^9^ Faculty of Environmental Sciences and Natural Resource Management Norwegian University of Life Sciences Ås Norway; ^10^ Natural Resources Institute Finland (Luke) Oulu Finland

**Keywords:** Arctic tundra, bacterial communities, climate change, fungal communities, ITEX, nitrogen cycling, warming

## Abstract

Climate warming is changing tundra vegetation in the Arctic, with implications for plant litter properties. Warming may thus modify bacterial and fungal communities and their nitrogen (N) cycling capacity in the litter layer, which in turn can affect plant N availability. To address potential warming effects, we characterized the responses of bacterial and fungal communities and their genetically encoded capacity for inorganic N‐transformations in the litter layer, as well as ^15^N natural abundance in the underlying soil layer as an integrated measure of N processes in the soil, in 16 long‐term alpine and Arctic tundra warming experiments distributed across 12 circumpolar locations. Although abundance, diversity, and composition of microbial communities were structured by the local conditions rather than experimental warming, warming indirectly modified microbial communities and their capacity for N transformations through changes in litter quality. Specifically, experimental warming resulted in stronger connections between the capacity for nitrification, denitrification and N‐fixation in the litter and the δ^15^N signature in the soil. These warming‐induced connections were mainly mediated by increased dominance of herbs but also increased litter mass. These findings suggest accelerated inorganic N cycling in the litter layer with warming, particularly coupled to local abundance of herbs, which can create positive feedback on plant growth as well as ecosystem respiration. Thus, microbial communities in the litter may contribute to an intensification of ongoing vegetation shifts across the tundra biome.

## Graphical abstract

Climate warming affects tundra vegetation, with implications for plant litter and associated bacterial and fungal communities. This study examined litter bacterial and fungal communities and their nitrogen (N) cycling potential across 16 long‐term warming experiments in arctic and alpine tundra. While local conditions primarily shaped microbial communities, warming indirectly affected microbial N‐cycling potentials through changes in herb dominance and litter mass, resulting in enhanced links between litter N cycling potentials and δ^15^N in the underlying soil. Our results suggest that warming‐driven vegetation changes may intensify N‐cycling with potential positive feedbacks on plant growth and ecosystem respiration across the tundra biome.

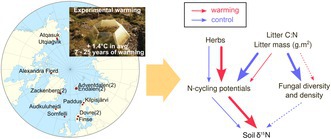

## 1 Introduction

The Arctic is experiencing faster climate warming compared to other regions (Meredith et al. 2019; Rantanen et al. 2022), leading to increased ecosystem respiration due to increases in both plant‐related and soil microbial respiration (Maes et al. 2024). Warming may stimulate N mineralization in tundra soils and other cold‐adapted ecosystems (Daebeler et al. 2017; Salazar et al. 2020), similarly to what is expected globally (Bai et al. 2013; Rustad et al. 2001). Warming is also causing increased growth and expansion of shrubs and graminoids into previous dwarf‐shrub or moss and lichen‐dominated tundra (Elmendorf et al. 2012; Myers‐Smith et al. 2011; Bjorkman et al. 2020). Such vegetation shifts and associated changes in plant leaf traits, together with increased amounts of litter, affect litter decomposition rates (Cornelissen et al. 2007; McLaren et al. 2017; Myers‐Smith et al. 2019). The expected increase in deciduous shrub and graminoid production will result in litter with lower carbon to nitrogen ratios (C:N), which generally promotes N mineralization (Buckeridge et al. 2013, 2010; Chu and Grogan 2010). Thus, warming can both directly and indirectly promote self‐reinforcing plant–soil feedback, by increasing litter decomposition and N‐mineralization and N‐availability, thereby supporting further plant growth (Buckeridge et al. 2010).

Litter decomposition comprises a continuum of organic and inorganic nutrient flows between plant and microbial biomass to soil (Berg and McClaugherty 2014; Cotrufo et al. 2013). Nitrogen flows in this continuum can be traced through changes in N isotopic signatures, which integrate the net effects of microbially mediated N transformations that fractionate N isotopes and thereby change the abundance of ^15^N relative to ^14^N (Robinson 2001; Dijkstra et al. 2008; Hobbie and Ouimette 2009). For example, when N is microbially transformed and lost from the soil, microorganisms preferentially use the lighter isotope, thereby enriching the residual soil N pool in ^15^N. Further, foliar and therefore litter ^15^N signatures vary with root‐associated symbiotic processes, for instance leading to larger ^15^N‐depletion in foliage of ericoid‐ and ectomycorrhizal shrubs relative to arbuscular‐ or non‐mycorrhizal herbs (Craine et al. 2009). Because plant litter is the main input of N to tundra soils, both litter characteristics and microbial N transformations occurring in the decomposing litter layer affect the ^15^N signature of the underlying soil (Craine et al. 2015). Thus, variation in soil ^15^N signatures (denoted δ^15^N) indicates shifts in N dynamics across the litter‐soil continuum. With warming, direct and indirect modifications of microbially driven N cycling in the litter layer can thus have cascading effects on soil δ^15^N and ultimately affect plant N uptake. Yet, microbial communities in litter layers are poorly described compared to soil communities.

A few studies have characterized fungal communities in litter layers of boreal forests (e.g., Bödeker et al. 2016; McGuire et al. 2010; Otsing et al. 2018), and Arctic tundra (Christiansen et al. 2017; Clemmensen et al. 2021). There are no reports of bacterial and inorganic N‐cycling communities in tundra litter layers, even though N cycling communities in soil were altered by warming (Deslippe et al. 2005; Walker et al. 2008). Further, inorganic N cycling capacity has been shown to be tightly coupled to vegetation shifts in the subarctic treeline ecotone (Clemmensen et al. 2021). Based on δ^15^N signatures, a recent study in Arctic and alpine tundra reported indications that warming altered N‐cycling in litter (Jeanbille et al. 2021). More knowledge about how microbial communities and their N‐cycling capacity in litter respond to warming and its cascading effects on N cycling in the litter‐soil continuum is needed, since alterations in litter N cycling could contribute to further vegetation changes and increased ecosystem respiration.

Our aim was to determine responses of litter microbial communities and their genetic potential for N cycling to climate warming across the tundra biome at the circumpolar scale. To achieve this goal, we sampled 16 long‐term warming experiments distributed across 12 locations. We followed an integrative view of litter processes and microbial communities across the plant litter–soil continuum (Cotrufo et al. 2013). More specifically, we examined both direct and indirect responses of bacterial and fungal communities as well as functional groups involved in inorganic N cycling in the litter layer to test the hypotheses that (i) warming increases the microbial capacity for inorganic N transformations in the litter layer due to increased N‐mineralization with warming (Daebeler et al. 2017; Salazar et al. 2020), (ii) warming effects on fungal and bacterial communities, in terms of their abundance, diversity, and composition, in the litter layer can be both direct and indirect, with the latter depending on local variation in vegetation, litter quality and quantity, and (iii) effects of vegetation properties on microbial N cycling capacities in the litter layer change under warmed conditions, which mediates changes in soil δ^15^N.

## 2 Material and Methods

### 2.1 Study Sites and Sample Collection

Litter and soil samples as well as vegetation surveys were obtained from 16 warming experiments at 12 alpine and Arctic tundra sites (Figure S1, Table S1). Each experiment employed a passive warming treatment using open‐top chambers (OTCs) that raise the mean summer air temperature by 1.4°C on average (Maes et al. 2024). The experiments were established between 1989 and 2007, with controls and OTCs established the same year within a site, and the duration of the warming experiments therefore differed among sites (Table S1). Site names were abbreviated with the first three letters of the geographic name, followed by “ev” for evergreen, “de” for deciduous, and “gr” for graminoids to indicate the dominant vascular plant functional group in the vegetation according to previous studies at the sites (Table S1). Sites in Endalen were named End_ev_cas and End_ev_dry for dominance by *Cassiope* and *Dryas*, respectively. The OTC and control plots are either randomly distributed at the experimental site or are paired in randomly distributed blocks, and in both cases with 4–5 replicates of each treatment.

Litter and soil were sampled from all sites during July and August of 2014, except for Alexandra Fiord in Canada which was sampled in August of 2015. Three cores, with a 3 cm diameter, were taken randomly from each of the control and OTC plots to a maximum depth of 20 cm, depending on the soil thickness. Samples were kept cool (< 4°C) but not frozen during transportation from the field to the laboratory in Uppsala and all samples arrived within a week of sampling. The litter, organic and mineral soil layers were separated for each of the three cores per plot, and material from the same layer was pooled (mineral soil was not included in this study). Larger stones and roots (> 5 mm diameter) were removed, and the material was weighed, homogenized, and stored at −20°C. Sub‐samples were weighed, freeze‐dried, and gravimetric water content (%) was determined after freeze‐drying. A total of 276 (16 sites × 2 treatments × 4–5 plots × 2 layers) freeze‐dried litter and organic soil samples were milled to fine powder and later subsampled for analyses. Subsamples (5–30 mg) were analyzed for total C and N content and ^15^N/^14^N ratio using an Isoprime isotope ratio mass spectrometer with continuous flow (Isoprime Ltd., Cheadle Hulme, UK) coupled to a Eurovector CN elemental analyzer (Eurovector SPA, Redavalle, Italy).

### 2.2 Vegetation Surveys and Litter Quality

**FIGURE 1:**
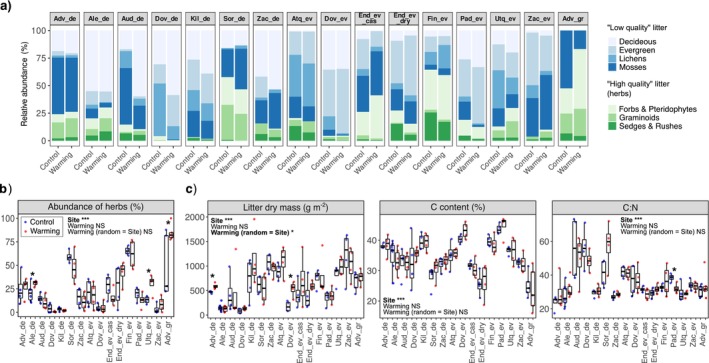
Plant functional groups and litter properties across sites and warming treatment. (a) Relative abundance of plant growth forms. (b) Relative abundance of herbs within and across sites. (c) Non‐collinear litter properties within and across sites. Boxes show the inter‐quartile range between the 1st and 3rd quartiles, with median indicated by the line and whiskers indicate the maximum and minimum of the inter‐quartile range. Site and treatment effects were tested using two‐way ANOVA, treatment means within sites were compared with Student's *t*‐test and treatment effect with site as random factor using linear mixed‐effect models (NS *p* > 0.05, *p < 0.05, **p < 0.01, ***p < 0.001).

Vegetation surveys were performed during the same summer as the soil and litter sampling, or up to 3 years earlier. Plant species cover was assessed by the point‐intercept method using 50–100 × 50–100 cm frames with 60–100 grid points (Molau and Mølgaard 1996). Single species abundance was calculated as a percentage of total plant intercepts (if plant intercepts > total points in frame) or of total points in frame (if plant intercepts < total points in frame). This means that we retained information on low cover vegetation, while total vegetation could never sum to more than 100%. This allowed us to capture potential relations between soil biota and variable total vegetation cover while decreasing biases due to a lack of multi‐layer records in some experiments. In three experiments, vegetation surveys were based on alternative methods; visually in subplots and either an average cover of plant species across 9 plots (dov_de) or the frequency of occurrence across 25 (sor_de) or 36 (fin_ev) subplots in each frame was calculated. Plant species were classified by growth forms and further assigned to functional groups, as a representation of their litter characteristics (Figure 1A, Table S2). Specifically, decomposability, assumed nitrogen content, and plant litter quality were considered for classification (i.e., both evergreen and deciduous shrubs were classified as low‐quality litter to reflect high lignin content of stems) (Cornelissen et al. 2007; Dorrepaal et al. 2005; Eskelinen et al. 2009). Together with shrubs, mosses and lichens were grouped as “low‐quality litter”, while forbs, pteridophytes (horsetails, clubmosses), grasses, sedges and rushes, were grouped as “high‐quality litter” (i.e., herbaceous plants, “herbs” litter Figure 1A, Table S2).

### 2.3 DNA Extraction and Sequencing of Fungal and Bacterial Communities in the Litter

DNA was extracted using the NucleoSpin Soil extraction kit (Macherey‐Nagel, Germany) from 50 mg of freeze‐dried and milled subsamples of litter (138 samples), following the manufacturer's instructions. The molecular weight was checked on agarose gel and quantified using the Nanodrop technology (Thermo Fisher Scientific, MA, USA) and Qubit fluorometer with the Qubit dsDNA BR kit (Life Technologies, CA, USA). Duplicate DNA extracts were pooled and stored at −20°C.

The fungal library was prepared following the protocol provided by Clemmensen et al. (2016), using one‐step amplification based on tagged primers with a unique multiplex primer pair for each sample. The forward primer gITS7, and a 3:1 mixture of the reverse ITS4 and ITS4a primers (Ihrmark et al. 2012; Sterkenburg et al. 2018; White et al. 1990) were used to target the ITS2 region between the 5.8S and 28S rRNA genes. The final concentrations in the PCR mix consisted of 0.5 and 0.3 μM for gITS7 and ITS4‐mix primers, respectively, 0.025 units μL^−1^ of DreamTaq polymerase, 200 μM dNTP and 750 μM MgCl_2_ in the DreamTaq buffer. PCR cycling consisted of a denaturation step for 5 min at 94°C, 30 amplification cycles of 30 s at 94°C, 30 s at 56°C and 30 s at 72°C, and a final extension step of 7 min at 72°C. Amplicons were pooled into two libraries after Qubit (Thermo Fisher) fluorometric dsDNA quantification and the size distribution was checked with Bioanalyzer (Agilent Tech, CA, USA). After ligation of adapters, sequencing was performed on eight PacBio PSII (Pacific Bioscience, CA, USA) SMRT cells per library.

The bacterial library was prepared using a two‐step amplification procedure of the V3–V4 region of the 16S rRNA gene using the pro341F and pro80imers (Takahashi et al. 2014). Each step was performed in duplicate and followed by electrophoresis and purification of the pooled duplicates with the AMPure PCR purification kit (Agencourt Bioscience Co, MA, USA). For the first amplification step, primers with Nextera adapters (Illumina Inc. CA, USA) were used at a final concentration of 0.25 μM, with 0.5 mg ml^−1^ BSA, 1 × Phusion High‐Fidelity PCR Master Mix (New England Biolabs, MA, USA) and 10 ng of template DNA in a 25 μL reaction. PCR cycling consisted of an initial denaturing step of 3 min at 98°C, followed by 25 cycles of 98°C for 30 s, 55°C for 30 s and 72°C for 30 s, and a final extension step of 10 min at 72°C. The second amplification step was performed with a diluted PCR product from the first step as template (1/10) and primers with Nextera tags, with a unique multiplex primer pair for each sample. The thermal cycling procedure was the same as in step 1, but with only 8 cycles and an extension step of 45 s. Amplicons were quantified with the Qubit fluorometer (Thermo Fisher Scientific) and the equimolar pool of amplicons was checked with the DNA kit of the Agilent 2100 Bioanalyzer (Agilent Tech) before sequencing using MiSeq (Illumina Inc.) using the second version of the 2 × 250 paired‐end chemistry.

### 2.4 Processing of ITS and 16S rRNA Gene Sequences

The fungal ITS raw reads (787,787) were processed using the SCATA pipeline (https://scata.mykopat.slu.se), which provides quality control and single‐linkage clustering for ITS data. The clustering similarity threshold was set to 98.5%, considering at least 85% of the longest sequence to align, with mismatch and gap extension penalties of 1 (Usearch, Edgar 2010). A total of 3121 fungal operational taxonomic units (OTUs) were kept after discarding 365 Viridiplantae identified by blasting the ITS sequences against the NCBI database. The PROTAX tool implemented in PlutoF (Abarenkov et al. 2018) was used for taxonomic assignment with a probability threshold of 0.9, and with taxonomy checked further against the UNITE database (Nilsson et al. 2019).

The 16S rRNA gene reads were first processed using the FASTX‐toolkit (http://hannonlab.cshl.edu/fastx_toolkit) for trimming, PEAR (Zhang et al. 2014) for merging, UCHIME (Edgar et al. 2011) for removing chimeras, and VSEARCH (Rognes et al. 2016) for dereplicating and clustering the sequences into OTUs at a 98% similarity threshold. Clusters with fewer than 3 reads were discarded, and original reads were mapped back to OTUs. Finally, the SILVA Incremental Aligner (SINA, Pruesse et al. 2012) was used to align and classify the resulting OTUs with the SILVA 138 database as a reference. Sequences identified as mitochondria and chloroplasts were discarded, resulting in 13,990 OTUs.

### 2.5 Quantitative PCR

The bacterial and fungal abundances in the litter samples were assessed by quantitative real‐time PCR (qPCR) of the ITS2 and the 16S rRNA gene using the same primers as for sequencing and this data was obtained from Jeanbille et al. (2021). The genetic capacity for inorganic N cycling was determined by qPCR using primers and amplification conditions listed in Table S3 for the following processes: denitrification by targeting the functional genes *nirS* (Michotey et al. 2000; Throbäck et al. 2004) and *nirK* (Henry et al. 2004), dissimilatory nitrate reduction to ammonium (DNRA) targeting *nrfA* (Mohan et al. 2004; Welsh et al. 2014), ammonia oxidation by archaea (AOA) and bacteria (AOB) targeting *amoA* (Rotthauwe et al. 1997; Tourna et al. 2011), and nitrogen fixation using *nifH* (Ando et al. 2005). All assays were performed twice on different runs in 15 μL reactions using the Biorad CFX Connect Real‐Time System (Bio‐Rad Laboratories, CA, USA). If the duplicates were different by one cycle (1 Cq) or more, a triplicate run was performed. The standard curves were obtained using serial dilutions of linearized plasmids holding the cloned fragments of the target gene. Each reaction contained 1× iQ SYBR Green Supermix (Bio‐Rad Laboratories), 0.5 μg μL^−1^ of BSA and 10 ng of template DNA. Primer concentrations were 0.25 μM for *nirK*; 0.5 μM for ITS2, 16S rRNA gene, *nrfA* and *amoA*; and 0.8 μM for *nirS* and *nifH*. Tests for PCR inhibition were performed for all samples by amplifying a known amount of the pGEM‐T plasmid (Promega, WI, USA) with the plasmid‐specific T7 and SP6 primers in the presence of 10 ng of template DNA or water. No inhibition was detected. ITS2 and 16S rRNA gene abundances were corrected by subtracting the proportion of plant or mitochondrial and chloroplast reads, respectively, retrieved from the sequence datasets, to obtain the final ITS2 and 16S rRNA gene counts.

### 2.6 Statistical Analyses

All statistical analyses were conducted using R statistical software version 3.6 (R Core Team 2018). For the calculation of alpha‐diversity indices, scaled ITS and 16S rRNA gene counts determined by qPCR for each sample were used as the total amount of sequences to rarefy the datasets. For community analysis, the data were not rarefied. Instead, Bayesian estimation of the sparse read counts using the CoDaSeq package (Gloor and Reid 2016) was done before a center log‐ratio transformation was performed with the zCompositions package (Palarea‐Albaladejo and Martín‐Fernández 2015) to account for the compositionality of the data (Gloor et al. 2017). To visualize the site variation based on log‐ratio transformed OTU abundances of the fungal and bacterial communities, principal component analyses (PCA) of euclidean distances were performed for each taxonomic group. The PCA axes were compared with random eigenvalues from the Broken‐stick model. To assess the sources of variation (i.e., litter amount and quality parameters) in the euclidean matrices of the log‐ratio transformed fungal and bacterial community data, we used a permutational multivariate analysis of variance (PerMANOVA) based on 10,000 permutations (McArdle and Anderson 2001) with the function adonis in the vegan package (Oksanen et al. 2013). As the effect of site was highly significant, the permutations were also constrained within each site using the strata option. The differences in relative abundances of the bacterial and fungal taxonomic groups between the warming treatment and control were assessed using linear mixed‐effect models (LMEM) based on log‐ratio transformed taxonomic abundances with site as a random factor, using the ARTool package, which allows the use of sparse and non‐normal data (Wobbrock et al. 2011). ARTool‐fitted LMEM were also done using rarefied abundances, which matched the log‐ratio‐based results, but led to more significant outputs. However, we only report the more conservative log‐ratio results. Following model fitting with ARTool, pairwise comparison of taxonomic group abundances between warming and control treatments were done using the emmeans package (Lenth 2018), which calculates the estimated marginal means (EMM, or least‐square means) and uses the Kenward‐Roger approximation for *F*‐test calculation.

Environmental variables, functional gene abundances and alpha diversity indices were tested for homoscedasticity and linearity using the Bartlett and Shapiro tests, respectively, and transformed (log, square root or Boxcox) when needed prior to statistical tests with linear assumptions. Environmental variables were checked for multicollinearity (Spearman Rho > 0.65, *p* < 0.05) and appropriate proxies for the full variable set were kept for further analysis (Table S4). Effects of warming and site on litter variables, 16S rRNA, ITS2 and N‐cycling gene abundances and alpha‐diversity indices were first determined by fitting two‐way ANOVA. Second, mixed‐effect models with site as a random factor were fitted using the lme4 package (Bates et al. 2015). Effects of non‐collinear litter parameters on 16S rRNA, ITS2 and N‐cycling gene abundances and alpha‐diversity indices were also determined by fitting mixed‐effect models. *p*‐values were calculated with the lmerTest package with default settings (Kuznetsova et al. 2017) or for interaction terms using a type III ANOVA with the Satterthwaite's method. In all cases, the model residuals were checked graphically and by using the Shapiro test. For a few models, one or two outliers leading to extreme residuals were removed to improve the linearity of the residuals. Student's *t*‐test was used to compare means between warming and control treatments at each site. Following mixed‐effect models, the slope contrasts between the control and warming conditions were assessed when the interaction between the warming treatment and the explanatory variable significantly affected the response variable (alpha‐diversity indices and N‐cycling genes). To calculate the contrasts, we used the emtrends command from the emmeans package (Lenth 2018), which computes the estimated marginal regression slopes and compares them using the Kenward‐Roger approximation of *F* tests. The significance level of the contrasts allowed us to compare the effects of the explanatory variables, depicted by the slopes, between the OTC and control plots.

### 2.7 Structural Equation Modelling

Structural equation modeling (SEM) was used to disentangle how warming modified the effects of litter amount and quality (C:N and herb cover) on N dynamics in the soil layer (using soil δ^15^N as a proxy for changes in soil N cycling) as mediated by fungal and bacterial diversity and N cycling capacity in the litter layer, thereby testing our third hypothesis. SEM allows description of multivariate relationships and testing of hypotheses by calculating simultaneous and sequential paths, considering both direct and indirect effects, among multiple drivers and the response variable of interest (Grace 2006). We tested and evaluated the relationships proposed in our meta‐model (Figure S2) with multi‐group SEM, by fitting the same model across both the warming and the control plots for comparison, using the lavaan package (Rosseel 2012). The hypotheses behind the different paths in the SEM are explained in the caption of Figure S2.

Before fitting the multi‐group SEM, variables were scaled to values from 0 to 1. To limit the number of variables included in the model, principal components combining quantitative microbial community attributes were obtained by including Pielou's evenness, observed richness and total abundance in PCAs for fungi and bacteria separately (Figure S3). In the SEM, non‐significant links and variables were pruned using the *modindices* command from the lavaan package, which estimates Chi‐square statistic improvements when variables are unconstrained. Moreover, to simplify the SEMs, variables (i.e., the beta‐diversity PCA axis, the bacterial attribute PCA axis (Figure S3), and the *nrfA* genetic potential) with no significant relationship to our main response variable, the soil δ^15^N, were pruned when no cascading effects were detected on intermediate parameters. Mediation tests were conducted by comparing the second order corrected Akaike's information criterion (AICc) for model selection of models, with and without additional paths. Global estimation and fitting of the model were done using maximum likelihood and chi‐square statistics, respectively. The models were evaluated by inspecting the root mean square error of approximation (RMSEA), the comparative fit index (CFI), and the standardized root mean square residual (SRMR). Control and warming models each included 66 samples with 8 free variables.

## 3 Results

### 3.1 Plant Growth Forms and Litter Properties Across the Warming Experiments

Composition of growth forms in the plant communities varied across the sites (Figure 1a). Experimental warming increased the abundance of herbs (high‐quality litter group) at the Alexandra Fiord site dominated by deciduous shrubs, the Utqiaġvik site dominated by deciduous shrubs, and the Adventdalen site dominated by graminoids, but when considering all sites, there was no warming effect on the abundance of herbs (Figure 1b). Yet, the duration of warming across experimental sites had a weak but significant effect on the composition of plant growth forms (PerMANOVA, R^2^ = 0.04). To reflect the litter layer amount and quality variation across sites, only the non‐collinear litter properties (dry mass (g m^−2^), C:N ratio and C content (%), Table S4) and herb abundance (Figure 1b) were included. Both herb abundance and C:N were indicators of litter quality, but they were not correlated (*p* > 0.05), thus they captured different aspects of litter quality (i.e., herbs are more indicative of available rather than total N). Warming did not affect these litter properties overall, but large differences were observed among sites (*p* < 0.001, Figure 1c). When controlling for the site effect, the litter dry mass was significantly higher in warmed plots (*F* = 4.54, *p* < 0.05). Warming effects were also observed within sites, with larger litter dry mass in the warmed plots at the Dovre site dominated by evergreen shrubs and at the Adventdalen site dominated by deciduous vascular plants, and with lower C:N ratios in warmed plots at the Paddus site dominated by evergreen shrubs (Student's *t*‐test, *p* < 0.05; Figure 1c). For litter C content, there were no effects of warming.

### 3.2 Microbial Abundances and Alpha‐Diversity

Large differences in bacterial and fungal abundances, richness and evenness were observed among the sites, but there were no overall effects of warming across sites (Figure S4). However, when the effect of the site was controlled for, bacterial evenness increased moderately and positively with warming (Table S5). Differences between warmed and control plots were observed at some sites and mainly attributed to fungal responses detected in the evergreen‐dominated sites, although not in a consistent manner. For instance, at the Endalen site dominated by the evergreen *Dryas*, fungal abundance and richness increased, and evenness decreased, whereas the opposite responses were observed at the Paddus site dominated by evergreens (Figure S4).

**FIGURE 2:**
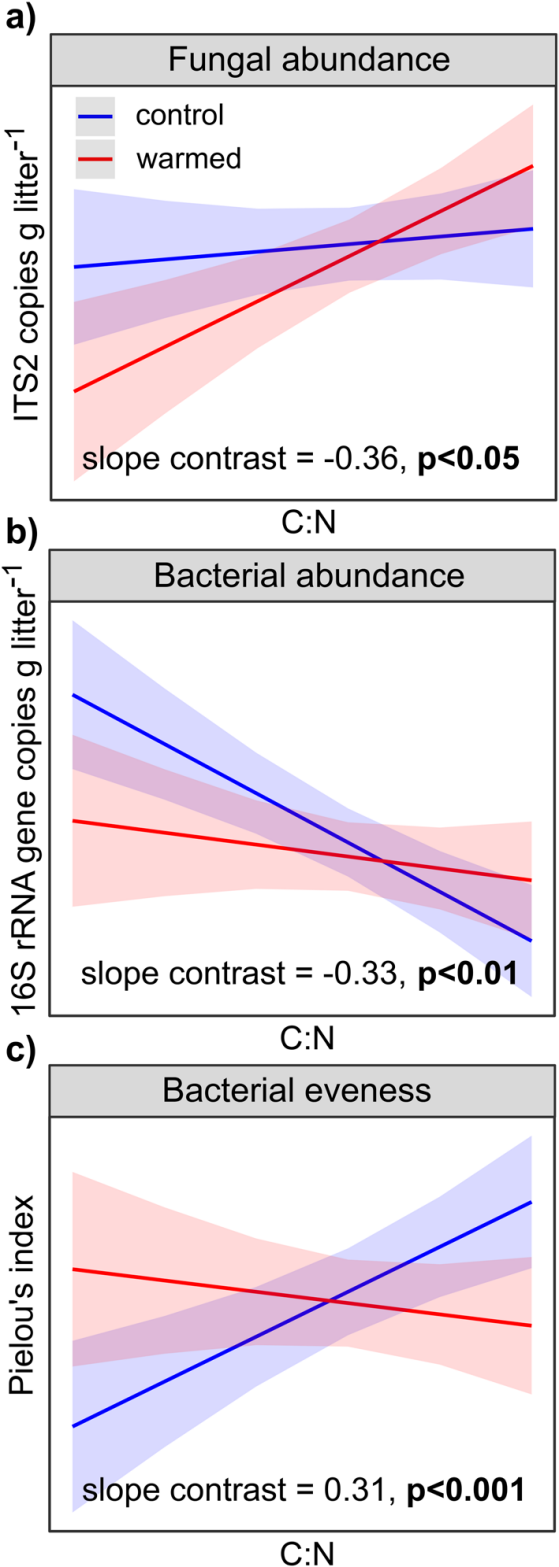
The relationship between litter C:N ratio and microbial factors in control and warmed plots. Relationships with (a) fungal abundance, (b) bacterial abundance, and (c) bacterial evenness. Estimated marginal slopes were computed on predicted values of the response variables based on linear mixed‐effect models using litter properties as predictors, with site as random factor. Only models including C:N are shown because they exhibited a significant interaction with warming (Table S5). Ribbons show the 95% confidence intervals. Because variables were transformed and scaled between 0 and 1 before fitting the models, neither data point nor scale are shown (i.e., all axis minimum is 0 and maximum 1).

Across all sites, fungal and bacterial abundances and richness were significantly correlated with litter mass and litter C:N ratio, and fungal abundance and richness were also significantly correlated with C content (Table S5). The warming treatment affected some of these relationships, as seen by significant interactions (slope contrasts) between the litter properties and microbial community characteristics (Figure 2, Table S5). In warmed plots, the fungal abundance was more positively correlated with C:N than in the control (Figure 2a), while the negative effect of increasing C:N on bacterial abundance in the control was lost with warming (Figure 2b). For evenness of the bacterial community, warming erased the positive effect of increasing C:N (Figure 2c). Thus, fungal abundance was more related to litter C:N in warmed plots, whereas bacterial abundance and evenness were significantly less controlled by litter C:N under warming.

### 3.3 Bacterial and Fungal Community Composition

**FIGURE 3:**
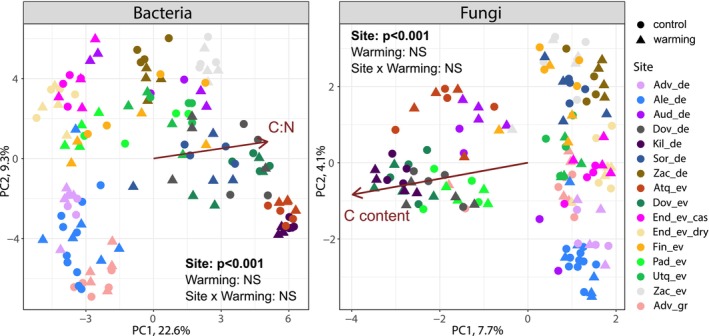
Bacterial and fungal community composition in litter samples across treatments and sites. The shape of the symbol denotes treatment, and sites are depicted by the color of the symbol. Principal component analyses are based on Euclidean distances of centered log‐ratio transformed OTU abundances. Unconstrained arrows show the direction and strength of litter variables that correlated significantly with the community composition according to PerMANOVA (*R* ^2^ = 5%, *p* < 0.001 and *R* ^2^ = 2%, *p* < 0.01 for C/N effect on bacterial communities and C content (%) effect on fungal communities, respectively). The non‐significant effect of warming (NS) and the significant effect of site were tested with PerMANOVA.

The bacterial and fungal communities varied more by site than between warming and control within site (Figure 3; *p* < 0.001), as reflected by the differences in taxonomic composition (Figure S5), and no significant effect of the warming treatment was observed (Figure 3, PerMANOVA *p* > 0.05). The bacterial communities were structured by litter C:N and fungal communities by C content (Table S6). To explore the effect of experimental warming on the environmental response of the communities, the communities in control and warmed plots were also analyzed independently (Table S6). This showed that control and warmed systems had contrasting responses to litter properties, with C:N significantly correlated with the structure of both the fungal and bacterial communities in warmed, but not in control plots, and C content significantly correlated with bacterial communities in control plots (Table S6). The effect of herb abundance was stronger for both bacteria and fungi in warmed plots, as seen from the higher *F* values. The effects of elevation on the bacterial and fungal communities were tested by using a binary variable (< 1000 or > 1000 m), in order to avoid a confounding site effect. Both bacterial and fungal communities were significantly affected by elevation (PerMANOVA, *R* ^2^ = 13% and *R* ^2^ = 5%, respectively, *p* < 0.001) but the interactions with the warming treatment were not significant, suggesting that warming did not affect the communities differently in higher elevation sites.

When inspecting fungal taxonomic groups from the phylum to the genus level for differential abundances in control versus warming treatments, a few overall significant shifts of less abundant fungal orders, families or genera were detected, but no shift was detected at higher taxonomic levels (Figure S5, Table S7). By contrast, the bacterial phyla *Bacteroidota*, *Bdellovibrionota*, *Firmicutes*, and *Patescibacteria*, and the acidobacterial Subgroup 5 were significantly more abundant in warmed plots (Table 1). At the order level and below, bacterial taxa with differential abundances between control and treatment had a low relative abundance but a high occupancy for most of the taxa.

**TABLE 1:** Effects of warming and litter properties on the genetic potential for inorganic nitrogen cycling.

	*amoA*		*nifH*		*nirS*		*nirK*		*nrfA*	
	Model estimate	*F*	Model estimate	*F*	Model estimate	*F*	Model estimate	*F*	Model estimate	*F*
C:N	0.14	4.24**	0.17	0.2	−0.31	10.62***	0.14	6.64**	−0.15	5.07**
DW (g m^−2^)	0.31	6.69**	0.43	3.06*	0.3	8.99***	0.31	7.28***	0.1	0.27
C content (%)	−0.45	9.41***	0.27	9.31***	−0.13	0.05	−0.45	1.1	−0.32	10.6***
Warming	−0.24	2.61	0.03	0.27	−0.11	0.93	−0.24	6.49**	−0.03	0.13
Warming × C:N	0.19	1.22	0.13	3.64*	0.01	0.01	0.19	4.67**	−0.03	0.09
Warming × DW (g m^−2^)	−0.15	1.36	−0.11	2.44	−0.13	1.64	−0.15	0.45	−0.15	4.03*
Warming × C content (%)	0.25	3.14*	−0.16	0.2	0.22	3.86*	0.25	3.58*	0.2	5.81**

*Note:* Linear mixed effects of litter C:N, dry weight (DW), carbon (C) content, and their interaction with warming were tested on the abundance of the *amoA* genes for archaeal ammonia oxidation. *nifH* for nitrogen fixation. *nirK* and *nirS* for denitrification and *nrfA* for dissimilatory nitrate reduction to ammonium (all determined as copy numbers per g of litter DW) with site as a random factor. Model estimates are fixed effect estimates. *p*‐values and associated *F*‐values were computed with type III ANOVA with Satterthwaite's method.

* *p* < 0.01.

** *p* < 0.05.

*** *p* < 0.01.

### 3.4 Abundances of Inorganic N‐Cycling Genes

The quantification of the bacterial *amoA* gene was below the detection limit of 10 copies per reaction (approximately 50,000 copies per g of litter DW) in more than 75% of the samples and hence, was not considered further. Despite differences within some sites, the abundances of genes involved in inorganic N cycling in the litter layer showed no overall response to warming, whereas the variation across sites was highly significant for all genes (*p* < 0.001, Figure S6). When site was included as a random factor, the abundance of the *nirK* gene, involved in denitrification, was negatively affected by warming, and the abundances of genes involved in archaeal ammonia oxidation (*amoA*) and denitrification (*nirK* and *nirS*), and N_2_‐fixation (*nifH*) were positively affected by the amount of litter, and *nifH* also by the litter C content (Table 1). By contrast, the litter C content negatively affected the abundances of genes for archaeal ammonia oxidation (*amoA*) and DNRA (*nrfA*). Also, the C:N ratio negatively influenced the abundance of *nrfA* and *nirS*, whereas *amoA* and *nirK* were positively affected by the C:N ratio. The *nirK*‐type denitrifiers were positively related to warming, but the interactions with litter variables indicated that they were more negatively correlated with C:N and C content in the control than in the warmed plots (Figure S7), similar to the trend observed for the 16S rRNA gene abundance (reflecting the total bacterial community). Similarly, *nrfA* abundance was more negatively correlated with the C content in the control plots (Figure S7).

### 3.5 Microbial Mediation of Litter Properties Affecting Nitrogen Transformations in the Litter‐Soil Continuum

Litter and soil stable isotope signatures (δ^15^N) were related to each other across all sites (Spearman's rho 0.19 (*p* < 0.05)). In the soil layer, δ^15^N was 1.4‰–6.3‰ higher than in the litter, except at the Adventdalen site dominated by graminoids (Figure S8a). Soil δ^15^N was not significantly impacted by warming across all sites, and only one site (Sornfelli dominated by deciduous shrubs) exhibited significantly higher δ^15^N with warming (Figure S8b).

**FIGURE 4:**
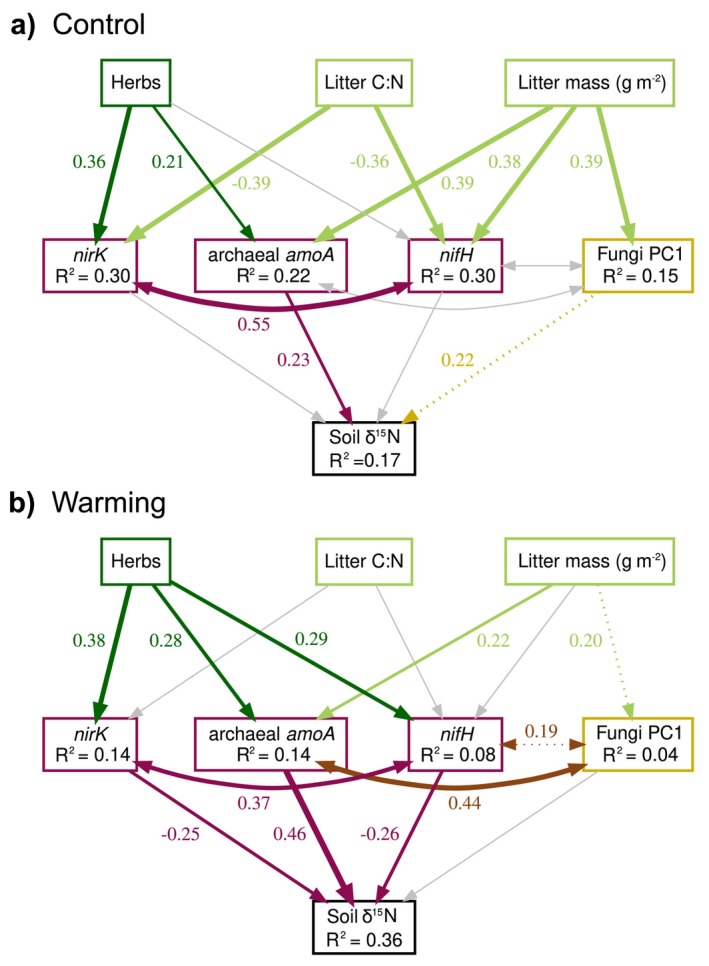
Structural equation models showing the influence of litter properties and microbial factors on soil N isotopic signature. Multi‐group structural equation models the links between herb abundance and litter properties (C:N ratio, dry weight (DW m^−2^)), microbial community components (fungal community attributes PC1, and the abundance of *nirK* for denitrification, *amoA* for ammonia oxidizing archaea, and *nifH* for nitrogen fixation, all expressed in copies g^−1^ DW litter) and soil N isotopic signature (soil δ^15^N) in control (a) and warmed (b) plots. PC1 reflects increasing fungal abundance and richness and decreasing fungal evenness (Figure S4A). Correlations are depicted by double arrows, regressions by one‐sided arrows, with width proportional to the indicated standardized estimate of the regression. The coefficient for the relationship is shown for significant relationships (dashed paths have *p* < 0.1, solid paths *p* < 0.05 and grey paths *p* > 0.1). Paths were pruned when not significant in at least one treatment. Standardized coefficients of the proportion of variation explained for each non‐exogenous variable are indicated in the boxes. The multi‐group model was robust with *χ* ^2^ = 23.70, with 25 df, *p* = 0.65, RMSEA = 0.000 (upper ci = 0.081, lower ci = 0.000), CFI = 1, and SRMR = 0.051.

Soil δ^15^N was used as the final response variable of a multigroup SEM to test our third hypothesis (Figure S2). The *nrfA* and *nirS* gene abundances and the bacterial community attributes (PC1, Figure S3b) were not significantly linked to the soil δ^15^N and were therefore pruned from the SEM model. Based on the SEM outcomes for ambient and warmed conditions (Figure 4), warming relaxed the correlations between litter properties (mass and C:N ratio) and N‐cycle gene abundances, as well as the fungal community attributes (PC1 indicating higher fungal abundance and richness and lower evenness, Figure S3a). With warming, the genetically encoded bacterial capacity for inorganic N cycling and the fungal attributes were thus less explained by the model (all had lower *R* ^2^). However, the abundance of herbs was more strongly coupled to *nirK*, archaeal *amoA* and in particular *nifH* gene abundances in the warmed plots. Warming further increased the effects of these genes on soil δ^15^N, while the direct effect of fungal attributes disappeared. Fungal attributes instead became highly positively correlated with archaeal *amoA* gene abundances in warmed plots. The proportion of variation in δ^15^N explained by the model more than doubled (36%) in warmed conditions in comparison to the control (17%).

## 4 Discussion

There were few overall, direct effects of long‐term warming on the relative abundance of plant growth forms, plant litter properties, fungal and bacterial communities in litter, and their inorganic N‐cycling capacity across Arctic and alpine tundra, although some site‐specific responses to warming were observed. Thus, our results did not support the first hypothesis that warming would result in a general increase in the genetically encoded capacity for inorganic N transformation processes in litter layers. Instead, the environmental dependencies of the microbial communities and their N cycling capacity in the litter layer differed between warmed and control plots. This in turn resulted in changes in soil δ^15^N, suggesting that warming leads to altered soil N cycling.

Both bacterial and fungal community composition was more structured by the litter C:N ratio and herb abundance across warmed than control plots when accounting for the differences among sites. Since neither of these litter quality indicators was directly affected by warming, this suggests that warming increased the importance of local vegetation in controlling microbial communities. This could be due to spatial heterogeneity in plant community composition and related litter quality per se, but it could also be mediated through varied vegetation responses to warming. Thus, our results partly confirm our second hypothesis by showing that warming can exert indirect effects on litter microbial and fungal communities linked to local variation in the quality of litter. As such, litter C:N ratio became more strongly and positively related to fungal, but not bacterial, abundance under warmed conditions, suggesting that fungi obtained an advantage in lower quality litter (Eskelinen et al. 2009). In contrast to Deslippe et al. (2012), we observed a warming‐driven homogenization of bacterial evenness and abundance over the litter C:N gradient, which indicates that the bacterial dependence on low C:N was relaxed with warming. Hence, across this wide gradient of tundra ecosystems, bacteria did not become more abundant in N‐rich litter with warming, although this could be the case at specific sites (Eskelinen et al. 2009; Deslippe et al. 2012; Jeanbille et al. 2021). Low quality litter had both positive and negative effects on the abundances of N‐cycling guilds, while the amount of litter, which overall increased with warming, positively affected the abundance of functional guilds involved in ammonia oxidation, denitrification and nitrogen fixation. Nevertheless, the shifts in N‐cycling capacity depended less on litter C:N and mass, and more on the relative abundance of herbs. These findings suggest that N cycling may be coupled to an increased decomposition of high‐quality litter. Fungal richness and density in litter were more related to nitrogen fixation and ammonia oxidation capacity with experimental warming, which potentially illustrate alleviation of N limitation by fungal decomposition (Sistla et al. 2012), or alternatively, increased decomposition and N‐mineralization with warming (Maes et al. 2024; Salazar et al. 2020). In control plots, the negative association of litter C:N with capacity for denitrification (*nirK* type denitrifiers) reflects the negative relationship commonly observed between N‐mineralization and C:N in the tundra (Buckeridge et al. 2010; Chu and Grogan 2010), but with warming the constraint of C:N was alleviated. Overall, these indirect effects of warming indicate that the climatic effect on litter microorganisms is mainly mediated by the plant community.

Warming strengthened the relation between the microbial N‐cycling capacity in the litter layer and the δ^15^N signature of the underlying soil. This effect was due to differences in the plant community, which provides support for our third hypothesis that modifications in litter quality and quantity result in changes in soil δ^15^N, mediated by an alteration of the microbial N cycling capacities. The direct effect of herb abundance on soil δ^15^N was not significant in the SEM, suggesting that the foliar δ^15^N had no direct effect on the soil δ^15^N. Soil δ^15^N signatures are the result of the transformation of N compounds by microbial activities with different degrees of fractionation between N isotopes (Robinson 2001; Dijkstra et al. 2008; Hobbie and Ouimette 2009). Enzymatic processes preferentially transform molecules with the lighter ^14^N isotope over those with the heavier ^15^N isotope, which consequently build up in the soil organic matter accumulating in the underlying soil. In particular, the abundance of ammonia oxidizing guilds (*amoA*; the first step of the nitrification process that transforms ammonia to nitrate) contributed positively to the δ^15^N increase in the soil. Nitrification fractionates strongly against ^15^N (Robinson 2001), and ammonium dominates the low‐molecular weight N pool in tundra soil (Koranda and Michelsen 2024). Increased ammonia oxidation would thus explain the ^15^N enrichment of ammonium in tundra soils (Liu et al. 2018), while nitrate, to a higher degree, is taken up by plants or lost through leaching or denitrification, and the observed positive relation between ammonia oxidizers and soil δ^15^N could suggest an incorporation of residual ammonium into stable pools. The capacity for ammonia oxidation was dominated by archaeal ammonia oxidizers over the bacterial counterpart, similar to what was previously shown in Arctic soils (Alves et al. 2013). Because nitrogen fixation fractionates N isotopes to a lesser extent (Unkovich 2013), the observed negative relationship between N‐fixing capacity (*nifH*) and the δ^15^N of soil in warmed plots suggests that a higher N fixation rate in litter is related to lower soil δ^15^N, likely because the fixed N has a relatively low δ^15^N. A higher warming‐associated N fixation capacity in the litter layer would increase the substrate availability for archaeal ammonia oxidation leading to greater ammonia oxidation capacity (*amoA*) with warming (Daebeler et al. 2017), which would further contribute to the ^15^N enrichment of the soil. With warming, the denitrification capacity (*nirK*) became negatively related to the soil δ^15^N signature, despite the high isotopic fractionation of denitrification that should lead to a positive relation (Robinson 2001; Hobbie and Högberg 2012). This suggests efficient transformation of both ^15^N depleted and enriched nitrate in the litter layer by the denitrifiers, thus restricting ^15^N build‐up of N from this source in the underlying soil layer, as previously demonstrated in other biomes (Houlton et al. 2006). Higher nitrification activity, resulting in a larger available pool of ^15^N depleted soil nitrate, could also contribute to the negative correlation between the genetic capacity for denitrification and δ^15^N. Although other soil processes and microbial communities could have affected the soil δ^15^N signatures, denitrification, archaeal‐driven nitrification, and N fixation have likely increased with long‐term warming, accelerating belowground inorganic N cycling (Salazar et al. 2020).

Despite a general lack of direct warming effects on the bacterial and fungal communities, some taxa were affected by warming. For the bacteria, taxa that decreased were mainly related to representatives previously isolated or detected in cold environments, e. g. *Deinococcus* (Lee et al. 2014; Yang et al. 2016), *Chtonomonas*, *Bryobacter* (Danilova et al. 2016), *Fimbriiglobus* (Nakai et al. 2012), *Ktedonobacter* (Kim et al. 2015), *Methylocella* (Dedysh et al. 2004), *Candidatus Ovatusbacter* (Nakai et al. 2012) or had a preference for low temperature, for example, *Fimbriimonas*, *Candidatus Xiphinematobacter* (Delgado‐Baquerizo et al. 2018). The abundance of *Bryobacter*, *Candidatus Solibacter*, *Chtonomonas* and *Bradyrhizobium* genera has earlier been found to be affected by warming within the dominating moss species at the Audkuluheidi site (Klarenberg et al. 2020, 2021). In addition, possible plant pathogens from the actinobacterial order *Propionibacteriales*, and from the *Rhodococcus* and *Streptomyces* genera, increased with warming. The fungal taxonomic composition was more heterogeneous across sites and the assessed environmental variables were less important for the structuring of the fungal community. This implies an increased contribution of stochastic processes for fungal community assembly (Evans et al. 2017), at least at this scale of sampling. Within sites, some abundant fungal orders shifted with experimental warming, but across all sites only lowly abundant, albeit frequent, taxa differed significantly between treatments. For example, lichen biotrophs from the *Peltigerales* order declined with experimental warming, which may reflect the decline of lichen species in specific sites (Elmendorf et al. 2012). Parasitic yeasts like *Cystobasidium* or the parasitic/saprotroph order *Hypocreales*, and the family *Trichocomaceae* (*Eurotiales*), which is mostly represented by opportunistic saprotrophs, increased with warming. Shifts in these fungal taxa agree with earlier reports on tundra soils (Semenova et al. 2015; Geml et al. 2015; Deslippe et al. 2012), indicating some similar responses in litter and soil. However, most of the litter fungal community members were not affected by warming but rather varied in relation to vegetation differences across sites.

In conclusion, the fungal and bacterial community composition, diversity, and abundance in the litter layer in tundra ecosystems were structured by local conditions rather than experimental warming. This underlines the importance of multi‐site comparisons to identify broadly generalizable responses to long‐term warming of Arctic and alpine tundra (Metcalfe et al. 2018). Instead, warming exerted indirect effects on the microbial communities mediated by increased litter quantity and quality. These effects were likely due to the small‐scale heterogeneity of microbial responses to vegetation type driving litter quality, at least at the decadal timescales studied here. Using SEM, stronger linkages were identified across warmed plots between the genetic capacities for several inorganic N‐cycling processes in the litter and the δ^15^N signature of the underlying soil. This was positively influenced by the abundance of herbs that produce litter with higher decomposability, indicating that areas with more herbs are more prone to increased inorganic N‐cycling with warming. Increased inorganic N‐cycling capacity indicates higher availability of ammonium and nitrate, which can be assimilated by tundra plants (Liu et al. 2018; Sorensen et al. 2008). Increased N availability could create positive feedback on the growth of the aboveground plant community (Hicks et al. 2022), particularly plant species with high N demands that are already shifting with warming (Buckeridge et al. 2010). Increasing N fixation and inorganic N can also enhance soil organic matter decomposition, which is typically N‐limited in tundra ecosystems (Sistla et al. 2012) and thereby increase soil respiration (Maes et al. 2024). Overall, our results suggest that microbial communities performing crucial inorganic N transformations in the litter layer could contribute to the intensification of greening and vegetation shifts, and potentially organic matter decomposition, in the tundra biome.

## Author Contributions

**Mathilde Jeanbille:** data curation, formal analysis, investigation, methodology, visualization, writing – original draft, writing – review and editing. **Karina E. Clemmensen:** conceptualization, formal analysis, methodology, supervision, writing – original draft, writing – review and editing. **Jaanis Juhanson:** conceptualization, supervision, writing – review and editing. **Anders Michelsen:** investigation, writing – review and editing. **Juha Alatalo:** writing – review and editing. **Elisabeth J. Cooper:** investigation, writing – review and editing. **Greg H. R. Henry:** investigation, writing – review and editing. **Annika Hofgaard:** investigation, writing – review and editing. **Robert D. Hollister:** investigation, writing – review and editing. **Ingibjörg S. Jónsdóttir:** investigation, writing – review and editing. **Kari Klanderud:** investigation, writing – review and editing. **Anne Tolvanen:** investigation, writing – review and editing. **Sara Hallin:** conceptualization, funding acquisition, methodology, project administration, resources, supervision, writing – original draft, writing – review and editing.

## Conflicts of Interest

The authors declare no conflicts of interest.

## Supporting information


**Data S1:** Supporting Information.

## Data Availability

The data that support the findings of this study are openly available in Dryad (doi: 10.5061/dryad.3ffbg79z4). Raw sequences were deposited at the NCBI database under BioProject accession number PRJNA760312.
