# Supplementary material for: Warming‐Induced Effects on Microbial Communities and Nitrogen Cycling Capacity in Tundra Litter Are Modulated by Herb Abundance and Litter Quality
Source: Glob Chang Biol. 2025 Nov 12;31(11):e70582. doi: 10.1111/gcb.70582 (PMC12606403; doi:10.1111/gcb.70582)
Supplement: Supplementary file 1 — Data S1: Supporting Information. [file GCB-31-e70582-s001.pdf]

Supplementary Information

Warming-induced effects on microbial communities and N cycling capacity in tundra litter are modulated by herb abundance and litter quality

Jeanbille et al

Content:

Table S1. Experimental sites

Table S2. Vascular plant species and growth forms

Table S3. Primers and amplification conditions

Table S4. Variable proxies used in statistical analysis

Table S5. Linear mixed effects of litter properties, warming, and their interaction on bacterial and fungal abundances and diversity

Table S6. The effect of the litter properties and relative abundance of forbs, sedges, and grasses (herbs) on bacterial and fungal community composition

Table S7. Fungal and bacterial taxonomic groups in the litter samples that increased in the warmed or control plots

Figure S1. Map showing sampling locations

Figure S2. Structural equation meta-model

Figure S3. Principal component analyses of fungal and bacterial community attributes

Figure S4. Fungal ITS2 and bacterial rRNA gene abundances, and OTU richness and evenness

Figure S5. Relative abundances of bacterial and fungal taxonomic groups

Figure S6. Genetic capacities for inorganic nitrogen cycling

Figure S7. Contrasts between genetic capacity for N-cycling in relation to litter C content and C:N in control and warmed plots

Figure S8. Nitrogen isotope ratios ($\delta^{15}N$)

Table S1. Experimental sites and dominant plant, as described in the referenced literature. The abbreviated names were chosen according to the dominant vegetation type, with "de" indicating deciduous, "ev" evergreen, "dr" *Dryas*, "cas" *Cassiope*, and "gr" graminoid.

Full experimental site name	Abbreviated name	Latitude	Longitude	Elevation (m)	Duration of warming (years)	Dominant plant	Reference
Adventdalen wet	Adv_gr	78.1	16	50	11	*Dupontia fisherii*	Strebel et al. 2010
Adventdalen mesic	Adv_de	78.1	16	50	11	*Salix polaris*	Little et al. 2017
Alexandra Fiord willow	Ale_de	78.9	-75.8	30	23	*Salix arctica*	Hudson et al. 2011
Atqasuk dry heath	Atq_ev	70.29	-157.25	22	18	*Ledum palustre*	Hollister et al. 2015
Audkuloheidi 450m	Aud_de	65.14	-19.43	490	18	*Betula nana*	Jónsdóttir et al. 2005
Utqiaġvik dry heath	Utq_ev	70.19	-156.37	3	20	*Cassiope tetragona*	Hollister et al. 2015
Dovre dry	Dov_de	62.3	9.6	1090	16	*Betula pubescens*	unpubl. data; Hofgaard et al. 2010
Dovre upper tundra	Dov_ev	62.3	9.6	1090	16	*Empetrum nigrum*	unpubl. data
Endalen heath dryas	End_ev_dry	78.11	15.45	90	12	*Dryas octopetala*	Jónsdóttir et al. 2023
Endalen heath cassiope	End_ev_cas	78.11	15.45	90	12	*Cassiope tetragona*	Jónsdóttir et al. 2023
Finse heath dryas	Fin_ev	60.37	7.32	1550	14	*Dryas octopetala*	Klanderud et Totland 2007
Kilpisjärvi heath subarctic	Kil_de	69.47	20.45	670	20	*Betula pubescens*	Rinnan et al. 2009
Paddus heath	Pad_ev	68.21	18.49	450	25	*Empetrum nigrum*	Michelsen et al. 2012
Sornfelli dry alpine meadow	Sor_de	62	-7	600	13	*Salix herbaceae*	Fosaa et al. 2004
Zackenberg heath salix	Zac_de	74.3	-21	40	7	*Salix arctica*	Mosbacher et al. 2013
Zackenberg heath cassiope	Zac_ev	74.3	-21	40	7	*Cassiope tetragona*	Mosbacher et al. 2013

Table S2. Vascular plant species and their assigned growth forms and litter quality. In a few cases plants were only identified to genus.

Vascular plant species	Growth form	Litter quality
Agrostis canina	GRASS	High-quality litter
Agrostis capillaris	GRASS	High-quality litter
Alopecurus borealis	GRASS	High-quality litter
Andromeda polifolia	EVERGREEN	Low-quality litter
Antennaria dioica	FORB	High-quality litter
Anthoxanthum odoratum	GRASS	High-quality litter
Arctagrostis latifolia	GRASS	High-quality litter
Arctostaphylos alpinus	DECIDUOUS	Low-quality litter
Arctostaphylos uva-ursi	EVERGREEN	Low-quality litter
Armeria maritima	FORB	High-quality litter
Astragalus alpinus	FORB	High-quality litter
Astragalus frigidus	FORB	High-quality litter
Bartsia alpina	FORB	High-quality litter
Betula nana	DECIDUOUS	Low-quality litter
Betula pubescens ssp. tortuosa	DECIDUOUS	Low-quality litter
Calamagrostis stricta	GRASS	High-quality litter
Campanula rotundifolia	FORB	High-quality litter
Cardamine sp.	FORB	High-quality litter
Cardamine nymanii	FORB	High-quality litter
Carex atrata	SEDGE	High-quality litter
Carex bigelowii	SEDGE	High-quality litter
Carex capillaris	SEDGE	High-quality litter
Carex glareosa	SEDGE	High-quality litter
Carex parallela	SEDGE	High-quality litter
Carex rupestris	SEDGE	High-quality litter
Carex vaginata	SEDGE	High-quality litter
Cassiope tetragona	EVERGREEN	Low-quality litter
Cerastium sp.	FORB	High-quality litter
Cerastium alpinum	FORB	High-quality litter
Cerastium arcticum	FORB	High-quality litter
Comastoma tenellum	FORB	High-quality litter
Deschampsia flexuosa	GRASS	High-quality litter
Diapensia lapponica	EVERGREEN	Low-quality litter
Diphasiastrum alpinum	FORB	High-quality litter
Dryas integrifolia	EVERGREEN	Low-quality litter
Dryas octopetala	EVERGREEN	Low-quality litter
Dupontia sp.	GRASS	High-quality litter
Empetrum hermaphroditum	EVERGREEN	Low-quality litter
Empetrum nigrum	EVERGREEN	Low-quality litter
Epilobium anagallidifolium	FORB	High-quality litter
Equisetum arvense	PTERIDOPHYTE	High-quality litter
Equisetum scirpoides	PTERIDOPHYTE	High-quality litter
Equisetum variegatum	PTERIDOPHYTE	High-quality litter

Erigeron uniflorus	FORB	High-quality litter
Eriophorum scheuchzeri	SEDGE	High-quality litter
Euphrasia sp.	FORB	High-quality litter
Festuca sp.	GRASS	High-quality litter
Festuca brachyphylla	GRASS	High-quality litter
Festuca ovina	GRASS	High-quality litter
Festuca richardsonii	GRASS	High-quality litter
Festuca rubra	GRASS	High-quality litter
Festuca vivipara	GRASS	High-quality litter
Gentiana nivalis	FORB	High-quality litter
Hieracium alpinum	FORB	High-quality litter
Hierochloe alpina	GRASS	High-quality litter
Hierochloe odorata	GRASS	High-quality litter
Huperzia selago	PTERIDOPHYTE	High-quality litter
Juncus biglumis	RUSH	High-quality litter
Juncus trifidus	RUSH	High-quality litter
Juniperus communis	EVERGREEN	Low-quality litter
Kobresia myosuroides	SEDGE	High-quality litter
Koenigia islandica	FORB	High-quality litter
Ledum palustre	EVERGREEN	Low-quality litter
Linnaea borealis	FORB	High-quality litter
Loiseleuria procumbens	EVERGREEN	Low-quality litter
Luzula arctica	RUSH	High-quality litter
Luzula confusa	RUSH	High-quality litter
Luzula nivalis	RUSH	High-quality litter
Luzula spicata	RUSH	High-quality litter
Minuartia biflora	FORB	High-quality litter
Oxyria digyna	FORB	High-quality litter
Oxytropis lapponica	FORB	High-quality litter
Papaver radicatum	FORB	High-quality litter
Pedicularis sp.	FORB	High-quality litter
Pedicularis dasyantha	FORB	High-quality litter
Pedicularis hirsuta	FORB	High-quality litter
Pedicularis kanei	FORB	High-quality litter
Persicaria vivipara	FORB	High-quality litter
Phyllodoce caerulea	EVERGREEN	Low-quality litter
Pinguicula vulgaris	FORB	High-quality litter
Poa sp.	GRASS	High-quality litter
Poa alpigena	GRASS	High-quality litter
Poa alpina	GRASS	High-quality litter
Poa arctica	GRASS	High-quality litter
Poa glauca	GRASS	High-quality litter
Potentilla sp.	FORB	High-quality litter
Potentilla crantzii	FORB	High-quality litter
Potentilla hyparctica	FORB	High-quality litter
Pyrola norvegica	FORB	High-quality litter
Ranunculus acris	FORB	High-quality litter
Rhododendron lapponicum	EVERGREEN	Low-quality litter
Rumex acetosa	FORB	High-quality litter

Salix arctica	DECIDUOUS	Low-quality litter
Salix hastata	DECIDUOUS	Low-quality litter
Salix herbacea	DECIDUOUS	Low-quality litter
Salix phlebophylla	DECIDUOUS	Low-quality litter
Salix polaris	DECIDUOUS	Low-quality litter
Salix reticulata	DECIDUOUS	Low-quality litter
Salix rotundifolia	DECIDUOUS	Low-quality litter
Saussurea alpina	FORB	High-quality litter
Saxifraga sp.	FORB	High-quality litter
Saxifraga oppositifolia	FORB	High-quality litter
Saxifraga punctata	FORB	High-quality litter
Scorzoneroides autumnalis	FORB	High-quality litter
Selaginella selaginoides	PTERIDOPHYTE	High-quality litter
Silene acaulis	FORB	High-quality litter
Solidago virgaurea	FORB	High-quality litter
Stellaria crassipes	FORB	High-quality litter
Stellaria laeta	FORB	High-quality litter
Stellaria longipes	FORB	High-quality litter
Taraxacum sp.	FORB	High-quality litter
Thalictrum alpinum	FORB	High-quality litter
Tofieldia pusilla	FORB	High-quality litter
Trisetum spicatum	GRASS	High-quality litter
Vaccinium myrtillus	DECIDUOUS	Low-quality litter
Vaccinium uliginosum	DECIDUOUS	Low-quality litter
Vaccinium vitis-idaea	EVERGREEN	Low-quality litter
Veronica alpina	FORB	High-quality litter
Viscaria alpina	FORB	High-quality litter

Table S3. Primers and amplification conditions used for quantitative real-time PCR.

Target	Primer	Sequence	Amplification[a]	Reference[b]
16S	pro341F	CCTACGGGAGGCAGCAG	(95 °C 5 min)x1 (95 °C 15s. 55°C 30s. 72 °C 30s. 78 °C 10s)x35	Takahashi et al. 2014
	pro805R	GACTACNVGGGTATCTAATCC		Takahashi et al. 2014
ITS2	gITS7	GTGARTCATCGARTCTTTG	(95 °C 5 min)x1 (95 °C 15s. 56°C 30s. 72 °C 30s. 79 °C 5s)x35	Ihrmark et al. 2012
	ITS4	TCCTCCGCTTATTGATATGC		White et al. 1990
	ITS4a	TCCTCGCCTTATTGATATGC		Sterkenburg et al. 2015 (modified to cover Archeorizhomyces)
nirS	nirS_Cd3aF	AACGYSAAGGARACSGG	(95 °C 7 min)x1 (95 °C 15s. 65 °C 30s. 72 °C 30s) x5 (95 °C 15s. 60 °C 30s. 72 °C 30s. 80 °C 5s)x34	modified from Michotey et al.. 2000
	nirS_R3cd	GASTTCGGRTGSGTCTTSAYGAA		modified from Throbäck et al.. 2004
nirK	nirK_876F	ATYGGCGGVCAYGGCGA	(95 °C 7 min)x1 (95 °C 15s. 63 °C 30s. 72 °C 30s) x5 (95 °C 15s. 58 °C 30s. 72 °C 30s. 80 °C 15s) x24	Henry et al. 2004
	nirK_1040R	GCCTCGATCAGRTTRTGGTT		Henry et al. 2004
nrfA	nrfAF2aw	CARTGYCAYGTBGARTA	(95 °C 5 min)x1 (95 °C 15s. 57 °C 30s. 72 °C 30s) x6 (95 °C 15s. 52 °C 30s. 72 °C 35s. 80 °C 15s) x34	Welsh et al. 2014
	nrfAR1	TWNGGCATRTGRCARTC		Mohan et al. 2004
AOA *amoA*	CrenamoA23f	ATGGTCTGGCTWAGACG	(95 °C 5 min)x1 (95 °C 15s. 55 °C 30s. 72 °C 40s. 77°C 5s)x39	Tourna et al. 2011
	CrenamoA616r	GCCATCCATCTGTATGTCCA		Tourna et al. 2011
AOB *amoA*	amoA-1F	GGGGTTTCTACTGGTGGT	(95 °C 5 min)x1 (95 °C 15s. 55 °C 30s. 72 °C 40s. 77 °C 5s)x39	Rotthauwe et al. 1997
	amoA-2R	CCCCTCKGSAAAGCCTTCTTC		Rotthauwe et al. 1997
nifH	DVV	TIGCRAAICCICCRCAIACIACRTC	(95 °C 5 min)x1 (95 °C 15s. 58 °C 30s. 72°C 35s. 80 °C 5s)x39	Ando et al. 2005
	IGK3	GCIWTHTAYGGIAARGGIGGIATHGG IAA		Ando et al. 2005

[a] All protocols ended with a melt curve: (95°C 15s (60 to 95 °C 10s increment 0.5 °C) and fluorescence was acquired at 79°C

[b] In the reference list in the article

Table S4. Litter layer variable proxies used in statistical analysis. Proxies were determined based on multicollinearity analysis.

Proxies	Correlated parameters
Dry mass (g m^{-2})	C (g m^{-2})<br>N (g m^{-2})<br>fresh weight (g)<br>dry weight (g)<br>water content (%)<br>dry weight (%)<br>fresh weight (%)
C:N	N (%)
C (%)	None
Herbs	None

Table S5. Linear mixed effects of C:N, litter dry weight (DW), carbon (C) content (%) and their interactions with warming tested on bacterial and fungal abundances (16S rRNA gene and ITS copy numbers per g DW, respectively) and richness and evenness. Experimental site was considered as random factor, and p-values and associated F-values were computed using a type III ANOVA with the Satterthwaite's method (* $p < 0.05$, ** $p < 0.01$, *** $p < 0.001$).

	**Bacteria**									**Fungi**								
	Abundance			Observed richness			Pielou's eveness			Abundance			Observed richness			Pielou's eveness		
	Model estimate	F-value		Model estimate	F-value		Model estimate	F-value		Model estimate	F-value		Model estimate	F-value		Model estimate	F-value	
C:N	0.07	9.23	**	-0.30	8.34	**	0.20	0.70		0.07	5.99	**	0.17	5.40	*	-0.10	3.06	.
DW (g m^{-2})	0.28	11.16	**	0.28	10.52	**+	-0.05	2.31		0.28	7.63	*	0.43	19.78	***	-0.14	1.80	
C content (%)	0.21	0.20		-0.08	1.66		-0.03	0.05		0.21	3.87	.	0.27	4.27	*	0.01	0.10	
Warming	-0.14	1.91		0.01	0.00		0.18	4.08	*	-0.14	1.35		0.03	0.05		0.15	1.31	
Warming × C:N	0.33	5.39	*	0.09	0.57		-0.28	7.59	**	0.33	5.72	*	0.13	0.74		-0.18	1.42	
Warming × DW (g m^{-2})	-0.12	1.33		-0.08	0.78		-0.08	1.20		-0.12	1.31		-0.11	1.08		0.05	0.24	
Warming × C content (%)	-0.06	0.00		-0.05	0.30		0.02	0.08		-0.06	0.30		-0.16	1.72		-0.09	0.49	

Table S6. The effect of the litter properties and relative abundance of herbs (forbs, pterophytes, sedges, rushes, and grasses) on bacterial and fungal community composition across both treatments, and within each treatment. Each term was tested using univariate PerMANOVA (* $p < 0.05$, ** $p < 0.01$, *** $p < 0.001$). PerMANOVA models are based on the Euclidean matrices of centered log-ratio transformed bacterial and fungal OTU abundances. All models considered site as a random factor.

	**Bacteria**									**Fungi**								
	ALL			Control			Warmed			ALL			Control			Warmed		
	F.Model	R^2		F.Model	R^2		F.Model	R^2		F.Model	R^2		F.Model	R^2		F.Model	R^2	
**Litter parameters**																		
Dry mass (g m⁻²)																		
C content (%)	5.33	0.04	.	3.18	0.05	*	2.98	0.04	.	3.22	0.02	**						
C:N	7.57	0.05	***				3.72	0.05	**							1.68	0.03	*
**Herbs (%)**				1.85	0.03	*	1.97	0.03	*				1.5	0.02	*	1.74	0.03	***

Table S7. Fungal and bacterial taxonomic groups in the litter samples that increased in the warmed (red) or control plots (blue). Values in brackets indicate relative abundance (% of the whole dataset), followed by occupancy (% of individual sites) of the specific taxa across the whole dataset. Significantly shifting taxonomic groups were detected by linear mixed-effect models based on log-ratio transformed OTU abundances ($p<0.05$).

Phylum	Class	Order	Family	Genus
**Fungi**				
Ascomycota	Eurotiomycetes	Eurotiales (0.45. 74)	Trichocomaceae (0.45. 74)	
	Sordariomycetes	Hypocreales (1.26. 89)		
	Lecanoromycetes	Peltigerales	Lobariaceae (0.01. 4)	
			Peltigeraceae (0.19. 15)	
	Dothideomycetes	Pleosporales	Sporormiaceae (0.08. 32)	
		Capnodiales	Teratosphaeriaceae (0.01. 16)	*Oleoguttula* (0.01. 12)
Basidiomycota	Microbotryomycetes	Heterogastridiales	Heterogastridiaceae (<0.01. 4)	
	Tremellomycetes	Tremellales	Tremellaceae	*Dioszegia* (0.03. 38)
			Bulleribasidiaceae (0.74. 77)	*Vishniacozyma* (0.74. 77)
		Trichosporonales (0.03. 34)		
	Cystobasidiomycetes	Cystobasidiales (0.04. 43)	Cystobasidiaceae (0.04. 43)	*Cystobasidium* (0.04. 43)
Chytridiomycota	Chytridiomycetes	Spizellomycetales	Powellomycetaceae	*Powellomyces* (<0.01. 7)
Zygomycota	Mortierellales	Mortierellales (0.67. 87)		
**Bacteria**				
Acidobacteriota	Subgroup 5 (0.01. 86)			
	Acidobacteriae	Bryobacterales (0.82. 100)	Bryobacteraceae (0.82.100)	*Bryobacter* (0.82. 100)
		Subgroup 2 (0.15. 95.6)		
		Solibacterales	Solibacteraceae	Candidatus Solibacter (0.72. 100)
Actinobacteriota	Actinobacteria	Propionibacteriales (2.58. 100)	Nocardioidaceae (2.45. 100)	*Kribbella* (0.02. 63)
			Propionibacteriaceae (0.14.96)	
		Corynebacteriales	Nocardiaceae	*Rhodococcus* (0.11.81)
		Frankiales	Acidothermaceae (1.65. 99)	*Acidothermus* (1.65. 99)
			Pseudonocardiales	*Actinomycetospora* (0.12. 96)
		Kineosporiales	Kineosporiaceae	*Angustibacter* (0.01. 76)
				Kineococcus (0.06. 86)
		Micromonosporales	Micromonosporaceae	Luedemannella (0.08. 93)
		Streptomycetales	Streptomycetaceae	*Streptomyces* (0.16. 99)

Armatimonadota	Armatimonadia (0.40. 100)	Armatimonadales (0.40. 100)		
	Chthonomonadetes (0.25. 100)	Chthonomonadales (0.25. 100)	Chthonomonadaceae (0.25.100)	*Chthonomonas* (0.25. 100)
	Fimbriimonadia	Fimbriimonadales	Fimbriimonadaceae	*Fimbriimonas* (0.01. 74)
Bacteroidota (8.44. 100)	Bacteroidia	Cytophagales (1.68. 100)	Hymenobacteraceae (0.62. 99)	*Hymenobacter* (0.61. 99)
		Flavobacteriales (0.76. 97)	Flavobacteriaceae (0.69. 97)	*Flavobacterium* (0.69. 97)
		Sphingobacteriales	KD3-93 (0.01. 80)	
			Spirosomaceae (0.6. 99)	
			Sphingobacteriaceae	*Pedobacter* (1.04. 100)
		Chitinophagales	Chitinophagaceae	*Thermomonas* (0.01. 46)
	Kapabacteria	Kapabacteriales (<0.01. 71)		
Bdellovibrionota (0.24. 100)	Bdellovibrionia (0.12. 100)	Bacteriovoracales (0.03. 85)	Bacteriovoracaceae (0.03. 85)	*Peredibacter* (0.02. 78)
	Oligoflexia	Oligoflexales	Oligoflexaceae	*Oligoflexus* (0.03. 79)
Chloroflexi	TK10 (0.14. 98)			
	Ktedonobacteria	Ktedonobacterales	Ktedonobacteraceae	*Ktedonobacter* (0.06. 76)
Deinococcota (0.01. 58)	Deinococci (0.01. 58)	Deinococcales (0.01. 58)	Deinococcaceae (0.01. 58)	Deinococcus (0.01. 57)
Firmicutes (0.49. 100)	Clostridia	Clostridiales (0.33. 91)	Clostridiaceae (0.33. 91)	
Myxococcota	Bacteriap25 (<0.01. 66)			
	Polyangia	Haliangiales	Haliangiaceae	Haliangium (0.71. 100)
Patescibacteria (0.61. 100)	Saccharimonadia (0.60. 100)	Saccharimonadales (0.60. 100)	Saccharimonadaceae	TM7a (0.01. 63)
Planctomycota	Planctomycetes (0.17. 100)	Gemmatales (0.04. 99)	Gemmataceae (0.04.99)	*Fimbriiglobus* (<0.01. 67)
Proteobacteria	Alphaproteobacteria	Elsterales (0.09. 100)		
		Holosporales (0.01. 88)	Holosporaceae (0.01.88)	
		Rhodospirillales (0.03. 100)		
		Rhizobiales	Methylopilaceae (0.05. 80)	
			Rhizobiaceae	*Aureimonas* (0.31. 94)
				Neorhizobium (0.06. 84)
			Xanthobacteraceae	*Bradyrhizobium* (1.26. 100)
			Beijerinckiaceae	*Methylocella* (0.01. 93)
				Roseiarcus (0.55. 99)
			Incertae Sedis	*Phreatobacter* (0.01. 90)
		Caulobacterales	Caulobacteraceae	*Brevundimonas* (0.2. 91)
		Sphingomonadales	Sphingomonadaceae	Ellin6055 (0.03. 81)
		Acetobacterales	Acetobacteraceae	*Roseomonas* (0.16. 90)
	Gammaproteobacteria	JG36-TzT-191 (0.08. 93)		
		WD260 (0.57. 100)		
		Incertae Sedis	Unknown Family	*Acidibacter* (0.29. 100)

		Xanthomonadales	Rhodanobacteraceae	Candidatus Ovatusbacter (0.01. 91)
				Dyella (0.01. 31)
SAR324 clade (0.02. 92)				
Verrucomicrobiota	Verrucomicrobiae	Verrucomicrobiales (0.28. 100)	Rubritaleaceae (0.16. 96)	*Luteolibacter* (0.16. 96)
		Chthoniobacterales	Xiphinematobacteraceae (0.35. 100)	Candidatus Xiphinematobacter (0.35. 100)
		Pedosphaerales	Pedosphaeraceae	SH3-11 (0.01. 65) influencing

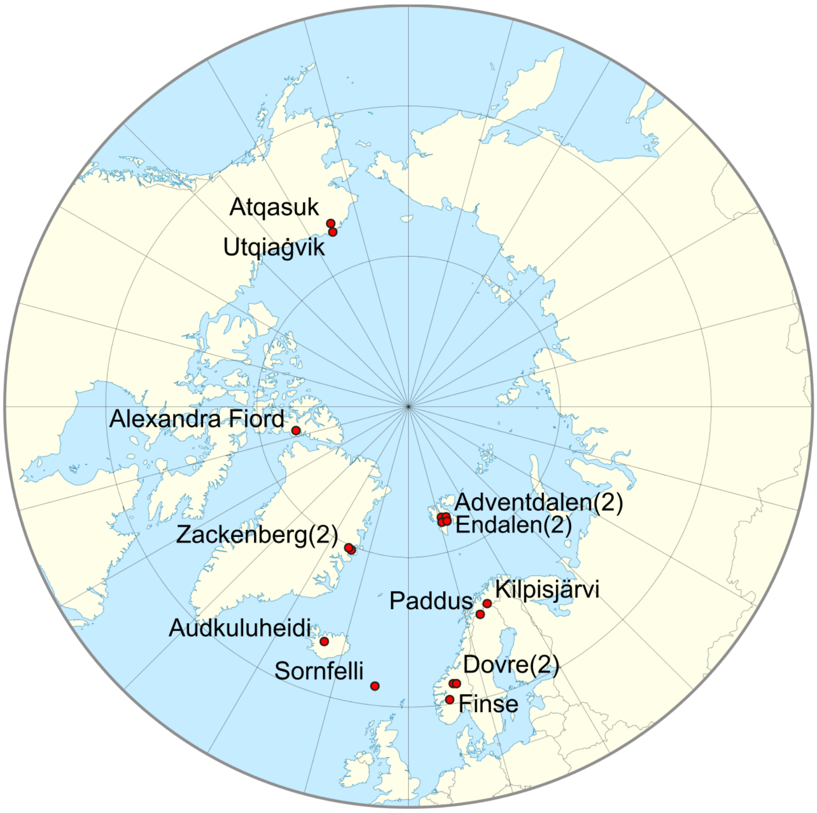

Figure S1. Map showing sampling locations. Among the 12 locations, four had two experimental sites (denoted by "(2)"), with 16 study sites in total. Map lines do not necessarily depict accepted national boundaries.

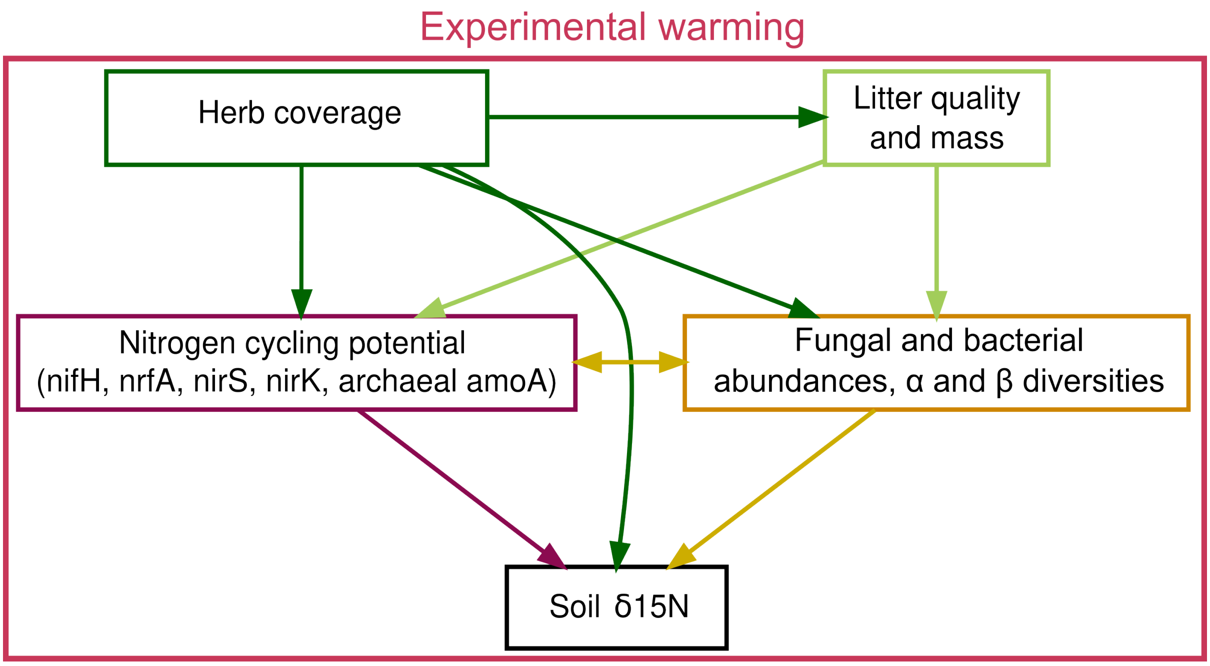

Figure S2. Structural equation meta-model showing theoretical links used for the SEM procedure. Experimental warming was assumed to influence the nature and intensity of the relationships mentioned hereafter. The genetic capacity for nitrogen (N) cycling, represented by abundances of nitrogen-fixing bacteria (*nifH*), bacteria performing dissimilatory nitrate reduction to ammonium (*nrfA*), denitrifiers (*nirS* and *nirK*), and archaeal ammonia oxidizers (*amoA*), was assumed to be controlled by herb cover and litter properties. Correlations between N-cycling capacity and the fungal community were allowed as fungi (both saprotrophs and root-associated) could compete for N with bacteria, and/or the fungal community and the N-cycling bacteria could benefit mutually from substrates released by the other. Correlations between the bacterial community and N cycling guilds were included since guild densities are related to the bacterial community composition, diversity and total abundance. Within the box for N cycling capacity, links between N-cycling potentials were allowed as correlations. The herb variable (high quality litter, negatively correlated to low quality litter, Fig. 1) was considered to directly influence litter quality and mass and the nitrogen fixation potential, as more symbiotic diazotrophs are expected to be present with more herbs. Soil $\delta^{15}N$ was expected to be influenced by the proportion of herbs (assuming the proportion of herbs could be correlated to root-driven processes and plant litter input signatures), bacterial and fungal abundances and community attributes, and by the litter N-cycling potentials. Fractionation during N-transformation processes in the litter layer is believed to affect the ^{15}N signature in the soil due to transport of different N species from the litter to the underlying soil. Thus, we used the soil $\delta^{15}N$ signature as a proxy for integrated effects of the different drivers in the litter layer.

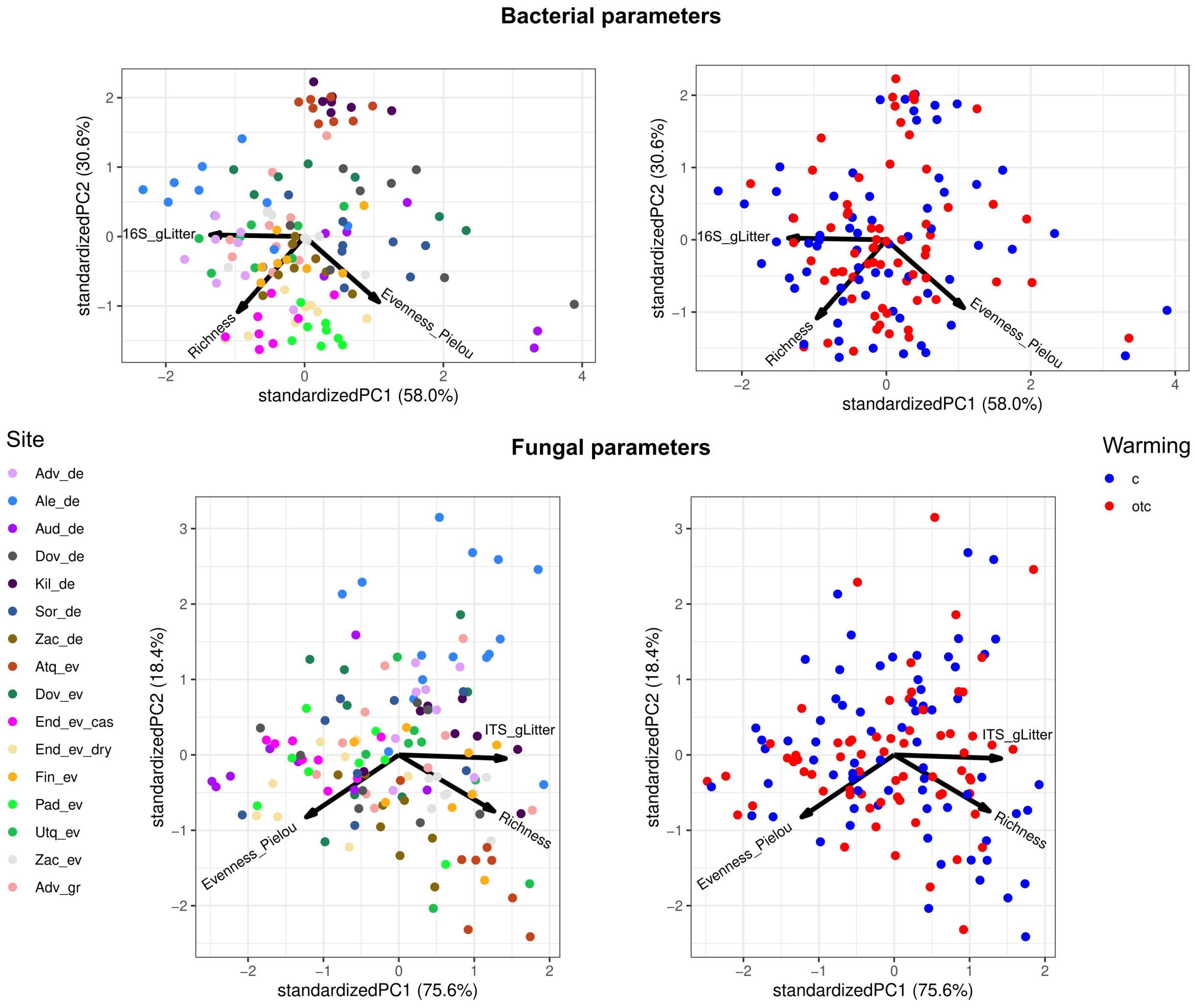

Figure S3. Principal component analyses of bacterial and fungal community attributes in litter samples across experimental sites (left side of the figure) and control and warmed plots (right side of the figure). Ordinations in the upper panels are based on bacterial abundance (16S rRNA copies g^{-1} litter DW), evenness (Pielou's) and richness. Ordinations in the lower panels are based on fungal abundance (ITS copies g^{-1} litter DW), evenness (Pielou's) and richness. Both fungal and bacterial PC1 were initially included in the SEM model, but the bacterial PC1 was later pruned from the model (see Methods).

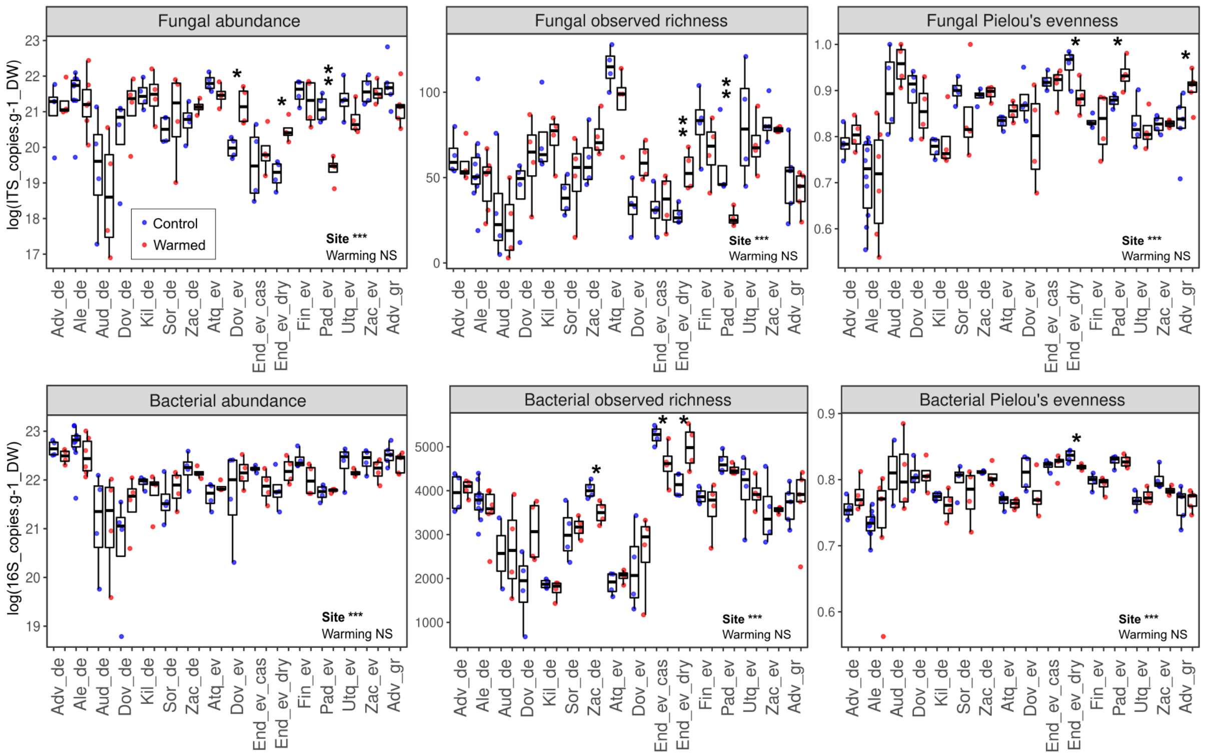

Figure S4. Fungal ITS2 and bacterial 16S rRNA gene abundances and observed OTU richness and evenness across sites. Boxes show the inter-quartile range between the 1st and 3rd quartiles. with median indicated by the line and whiskers indicate the maximum and minimum of the inter-quartile range. Site and warming effects were tested using two-way ANOVA and treatment means at each site were compared using Student's T test ($*$ $p < 0.05$, $**$ $p < 0.01$, $***$ $p < 0.001$).

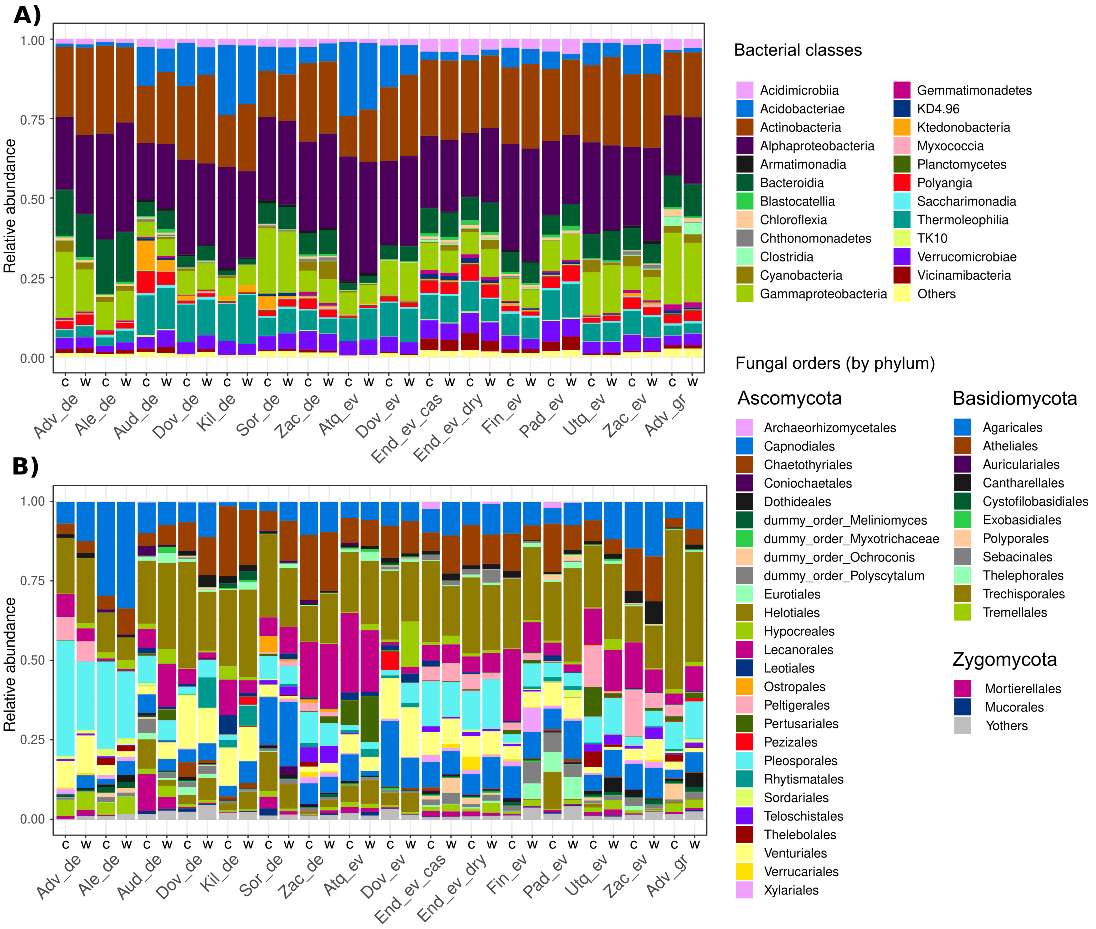

Figure S5. Relative abundances of **A)** bacterial and **B)** fungal taxonomic groups at the level of class and order, respectively, and classified with SINA 138 for bacteria and PROTAX for fungi. Those with relative abundance <0.05 % were grouped as "Others". For each experiment, the taxonomic composition is displayed for the control (denoted 'c') under the first bar followed by the warming (denoted 'w') under the second bar.

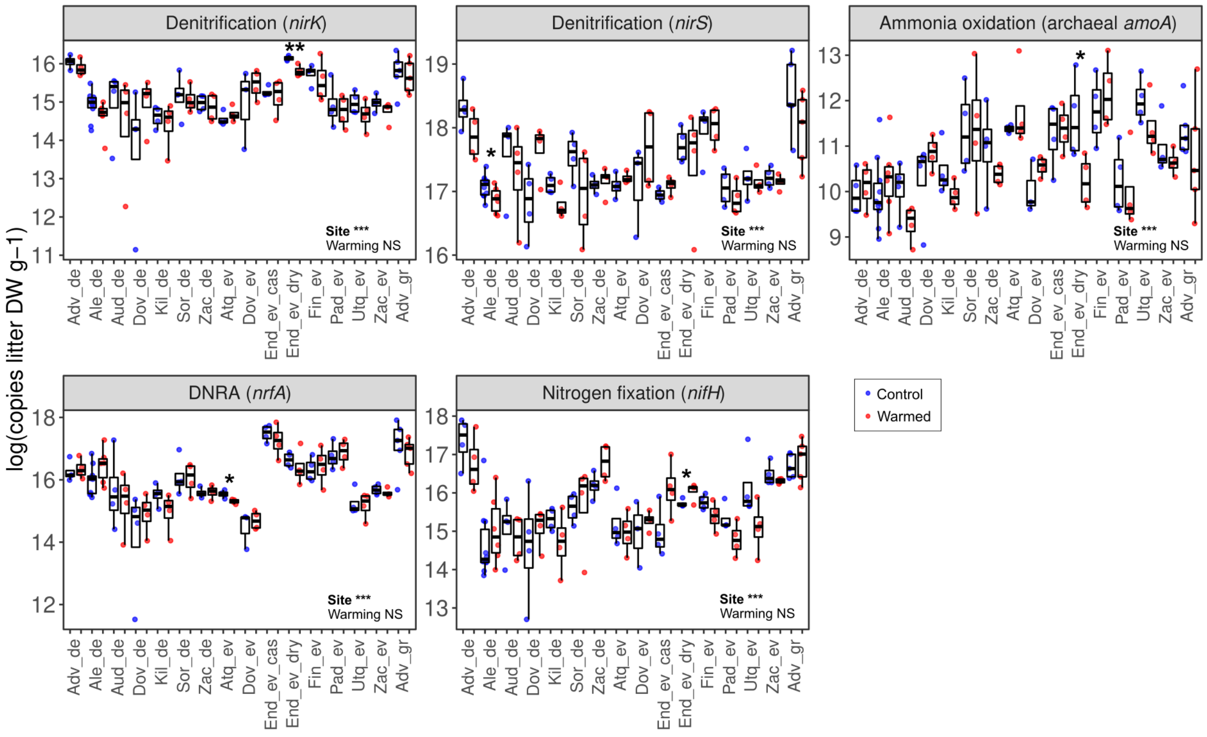

Figure S6. Genetic capacities for inorganic nitrogen cycling based on quantification of functional genes. Boxes show the inter-quartile range between the 1st and 3rd quartiles. with median indicated by the line and whiskers indicate the maximum and minimum of the inter-quartile range. Site and warming effects were tested using two-way ANOVA and treatment means at each site were compared using Student’s T test (∗ $p < 0.05$, ∗∗ $p < 0.01$, ∗∗∗ $p < 0.001$).

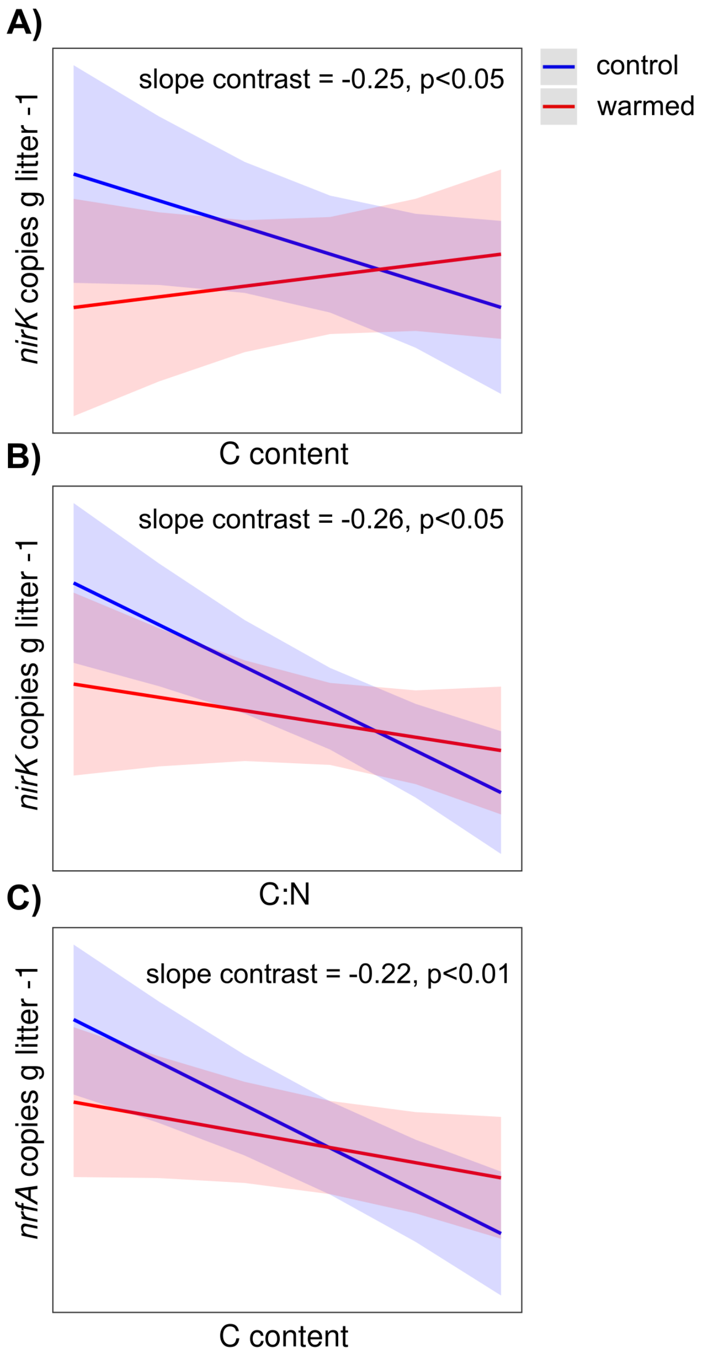

Figure S7. Contrasts between estimated marginal slopes of genetic capacity for denitrification (*nirK* copies g^{-1} litter DW) in relation to **A)** litter C content and **B)** litter C:N, and **C)** dissimilatory nitrate reduction to ammonium (DNRA, *nrfA* copies g^{-1} litter DW) in relation to C content in control and warmed plots. Ribbons show 95 % confidence intervals of the estimated marginal slopes. Estimated marginal slopes were computed on predicted values of the response variables based on linear mixed-effect models. Because variables were transformed and scaled between 0 and 1before fitting the models, neither data points nor scale are shown.

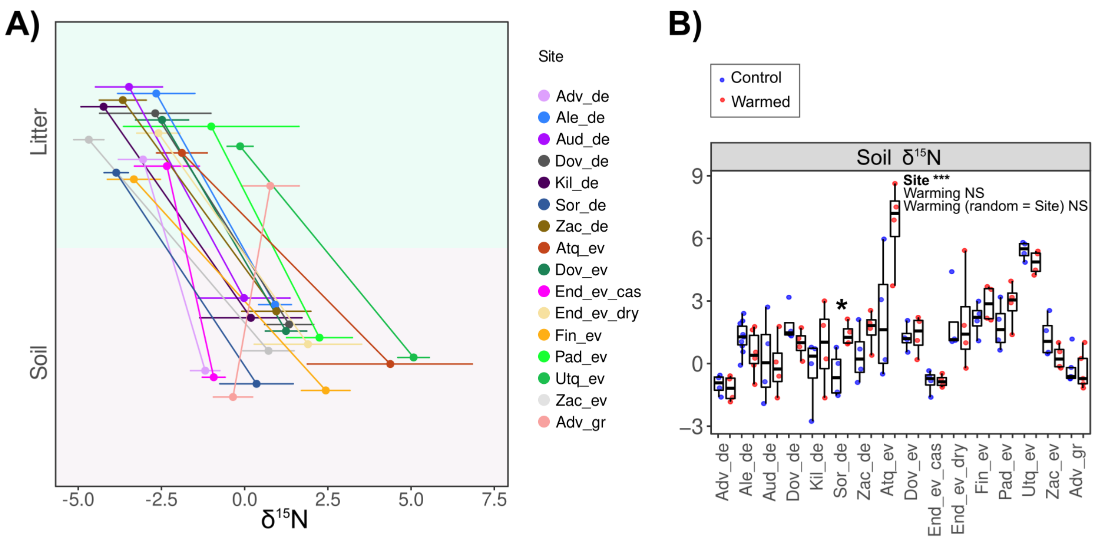

Figure S8. Nitrogen isotope ratios ($\delta^{15}N$). **A)** Site means across the litter-soil continuum, including samples from both warming and control. Error bars for each site indicate 95 % confidence intervals. **B)** Soil $\delta^{15}N$ comparison between warming and control treatments across sites. Boxes show the inter-quartile range between the 1st and 3rd quartiles, with median indicated by the line and whiskers indicate the maximum and minimum of the inter-quartile range. Site and warming effects were tested using one-way ANOVA, treatment means at each site were compared using Student's T test, and treatment effect with site as random factor was computed using a linear mixed-effect model ($*$ $p < 0.05$, $**$ $p < 0.01$, $***$ $p < 0.001$).

References

Ando, S., Goto, M., Meunchang, S., Thongra-ar, P., Fujiwara, T., Hayashi, H., & Yoneyama, T. (2005). Detection of *nifH* Sequences in Sugarcane (*Saccharum officinarum* L.) and Pineapple (Ananas comosus Merr.). Soil Science and Plant Nutrition, 51(2), 303–308. https://doi.org/10.1111/j.1747-0765.2005.tb00034.x

Fosaa, A.M., Sykes. M.T., Lawesson, J.E,. & Gaard, M. (2004). Potential effects of climate change on plant species in the Faroe Islands: Climate change on the Faroe Islands. Global Ecology and Biogeography, 13(5), 427-437. https://doi.org/10.1111/j.1466-822X.2004.00113.x

Henry, S., Baudoin, E., López-Gutiérrez, J.C., Martin-Laurent, F., Brauman, A., & Philippot, L. (2004). Quantification of denitrifying bacteria in soils by *nirK* gene targeted real-time PCR. Journal of Microbiological Methods, 59(3), 327–335. https://doi.org/10.1016/j.mimet.2004.07.002

Hofgaard, A., Løkken, J.O., Dalen. L., & Hytteborn, H. (2010). Comparing warming and grazing effects on birch growth in an alpine environment – a 10-year experiment. Plant Ecology & Diversity, 3(1), 18-27. https://doi.org/10.1080/17550871003717016

Hollister, R.D., J.L. May, K.S. Kremers, C.E. Tweedie, S.F. Oberbauer, J.A. Liebig, T.F. Botting, R.T. Barrett, and J.L. Gregory. (2015). Warming experiments elucidate the drivers of observed directional changes in tundra vegetation. Ecology and Evolution, 5(9). 1881–1895. https://doi.org/10.1002/ece3.1499

Hudson, J.M.G., Henry, G.H.R., & Cornwell, W.K. (2011). Taller and larger: Shifts in Arctic tundra leaf traits after 16 years of experimental warming: Arctic leaf trait response to

warming. Global Change Biology, 17(2), 1013-1021. https://doi.org/10.1111/j.1365-2486.2010.02294.x

Jónsdóttir, I.S., Magnusson, B., Gudmundsson, J., Elmarsdóttir, A. & Hjartarson, H. (2005). Variable sensitivity of plant communities in Iceland to experimental warming. Global Change Biology, 11(4), 553-563. https://doi.org/10.1111/j.1365-2486.2005.00928.x

Jónsdóttir, I. S., Halbritter, A.H., Christiansen, C.T., Althuizen, I.H.J., Haugum, S.V., Henn, J.J., Björnsdóttir, K., Maitner, B.S., Malhi, Y., Michaletz, S.T., Roos, R.E., Klanderud, K., Lee, H., Enquist, B.J. & Vandvik, V. (2023). Intraspecific trait variability is a key feature underlying high Arctic plant community resistance to climate warming. Ecological Monographs, 93(1), e1555. https://doi.org/10.1002/ecm.1555

Klanderud, K. & Totland, Ø. (2007). The relative role of dispersal and local interactions for alpine plant community diversity under simulated climate warming. Oikos, 116(8), 1279-1288. https://doi.org/10.1111/j.0030-1299.2007.15906.x

Little, C.J., Cutting, H., Alatalo, J. & Cooper, E.J. (2017). Short-term herbivory has long-term consequences in warmed and ambient high Arctic tundra. Environmental Research Letters, 12(2), 025001. https://doi.org/10.1088/1748-9326/aa579d

Michelsen, A., Rinnan, R. & Jonasson, S. (2012). Two decades of experimental manipulations of heaths and forest understory in the Subarctic. AMBIO, 41(3), 218–230. https://doi.org/10.1007/s13280-012-0303-4

Michotey, V., Méjean, V., & Bonin, P. (2000). Comparison of Methods for Quantification of Cytochrome cd_1-Denitrifying Bacteria in Environmental Marine Samples. Applied and Environmental Microbiology, 66(4), 1564–1571.

Mohan, S.B., Schmid, M., Jetten, M. & Cole, J. (2004). Detection and widespread distribution of the *nrfA* gene encoding nitrite reduction to ammonia, a short circuit in the biological nitrogen cycle that competes with denitrification. FEMS Microbiology Ecology, 49(3), Article 3. https://doi.org/10.1016/j.femsec.2004.04.012

Mosbacher, J.B., Schmidt, N.M. & Michelsen, A. (2013). Impacts of eriophyoid gall mites on arctic willow in a rapidly changing Arctic. Polar Biology, 36(12), 1735–1748. https://doi.org/10.1007/s00300-013-1393-6

Rinnan, R., Stark, S. & Tolvanen, A. (2009). Responses of vegetation and soil microbial communities to warming and simulated herbivory in a subarctic heath. Journal of Ecology, 97(4), 788-800. https://doi.org/10.1111/j.1365-2745.2009.01506.x

Rotthauwe, J. H., Witzel, K. P. & Liesack, W. (1997). The ammonia monooxygenase structural gene *amoA* as a functional marker: Molecular fine-scale analysis of natural ammonia-oxidizing populations. Applied and Environmental Microbiology, 63(12), 4704–4712. https://doi.org/10.1128/aem.63.12.4704-4712.1997

Strebel, D., Elberling, B., Morgner, E., Knicker, H.E. & Cooper, E.J. (2010). Cold-season soil respiration in response to grazing and warming in High-Arctic Svalbard. Polar Research, 29(1), 46-57. https://doi.org/10.1111/j.1751-8369.2010.00154.x

Throbäck, I.N., Enwall, K., Jarvis, Å. & Hallin, S. (2004). Reassessing PCR primers targeting *nirS*, *nirK* and *nosZ* genes for community surveys of denitrifying bacteria with DGGE. FEMS Microbiology Ecology, 49(3), 401–417. https://doi.org/10.1016/j.femsec.2004.04.011

Tourna, M., Stieglmeier, M., Spang, A., Könneke, M., Schintlmeister, A., Urich, T., Engel, M., Schloter, M., Wagner, M., Richter, A. & Schleper, C. (2011). *Nitrososphaera viennensis*, an ammonia oxidizing archaeon from soil. Proceedings of the National Academy of Sciences, 108(20), 8420–8425. https://doi.org/10.1073/pnas.1013488108

Welsh, A., Chee-Sanford, J.C., Connor, L.M., Löffler, F.E. & Sanford, R.A. (2014). Refined NrfA Phylogeny Improves PCR-Based *nrfA* Gene Detection. Applied and Environmental Microbiology, 80(7), 2110–2119. https://doi.org/10.1128/AEM.03443-13.
